# Integrating full-length transcriptomics and metabolomics reveals the regulatory mechanisms underlying yellow pigmentation in tree peony (*Paeonia suffruticosa* Andr.) flowers

**DOI:** 10.1038/s41438-021-00666-0

**Published:** 2021-11-01

**Authors:** Xiaoning Luo, Daoyang Sun, Shu Wang, Sha Luo, Yaqi Fu, Lixin Niu, Qianqian Shi, Yanlong Zhang

**Affiliations:** College of Landscape Architecture and Art, Northwest A&F University, Yangling, China

**Keywords:** Metabolomics, Gene regulation, Secondary metabolism, Transcriptomics

## Abstract

Tree peony (*Paeonia suffruticosa* Andr.) is a popular ornamental plant in China due to its showy and colorful flowers. However, yellow-colored flowers are rare in both wild species and domesticated cultivars. The molecular mechanisms underlying yellow pigmentation remain poorly understood. Here, petal tissues of two tree peony cultivars, “High Noon” (yellow flowers) and “Roufurong” (purple–red flowers), were sampled at five developmental stages (S1–S5) from early flower buds to full blooms. Five petal color indices (brightness, redness, yellowness, chroma, and hue angle) and the contents of ten different flavonoids were determined. Compared to “Roufurong,” which accumulated abundant anthocyanins at S3–S5, the yellow-colored “High Noon” displayed relatively higher contents of tetrahydroxychalcone (THC), flavones, and flavonols but no anthocyanin production. The contents of THC, flavones, and flavonols in “High Noon” peaked at S3 and dropped gradually as the flower bloomed, consistent with the color index patterns. Furthermore, RNA-seq analyses at S3 showed that structural genes such as *PsC4H*s, *PsDFR*s, and *PsUFGT*s in the flavonoid biosynthesis pathway were downregulated in “High Noon,” whereas most *PsFLS*s, *PsF3H*s, and *PsF3’H*s were upregulated. Five transcription factor (TF) genes related to flavonoid biosynthesis were also upregulated in “High Noon.” One of these TFs, PsMYB111, was overexpressed in tobacco, which led to increased flavonols but decreased anthocyanins. Dual-luciferase assays further confirmed that PsMYB111 upregulated *PsFLS*. These results improve our understanding of yellow pigmentation in tree peony and provide a guide for future molecular-assisted breeding experiments in tree peony with novel flower colors.

## Introduction

Tree peony (*Paeonia suffruticosa* Andr.) belongs to the section *Moutan* in the genus *Paeonia* of the family Paeoniaceae and is a traditional horticultural plant in China^1^. Floral color is one of the crucial ornamental characteristics of tree peony, which has more than 2000 cultivars with 9 major colors worldwide^2,3^. The flower colors of domesticated tree peony are generally white, pink, purple, or red, but few flowers are pure yellow. Currently, new tree peony cultivars with novel pure yellow corollas have become a breeding focus with great economic potential^4^. “High Noon,” a hybrid of *P*. *suffruticosa* × *Paeonia* *lutea*, is one of the most famous and popular tree peony cultivars. It possesses pure yellow, cup-shaped, and semidouble flowers. Due to its strong ecological adaptability, “High Noon” has been increasingly cultivated in the major production areas of tree peony around the world. However, the molecular basis of yellow coloration in tree peony remains elusive. Therefore, yellow-colored “High Noon” provided a good candidate for research on yellow coloration in tree peony flowers.

The chemical compounds determining flower colors mainly include flavonoids, carotenoids, chlorophylls, and their derivatives^5^. Flavonoids are the decisive pigment groups of most flower colors and are present in the vacuoles of petal epidermal cells^6^. In the flavonoid biosynthesis pathway, anthocyanins confer pink, red, purple, and blue colors to flowers and other organs, whereas chalcones and aurones are deep yellow, and flavones and flavonols are faint yellow or almost colorless. Carotenoids consist of carotenes and xanthophylls, which are present in plastids and can render plants yellow, orange, and red^5^. Chlorophylls are the predominant pigments in the green organs of plants, such as leaves and stems. Previous studies have found that tree peony flower pigments were mainly anthocyanins, including 3-*O*-glucosides and 3,5-di-*O*-glucosides of cyanidin, peonidin, and pelargonidin; flavones, including multiform glucosides of apigenin (Ap), luteolin (Lu), and chrysoeriol (Ch); and flavonols, including multiform glucosides of kaempferol (Km), quercetin (Qu), and isorhamnetin (Is)^7–10^. In eight different color series from white to red, to yellow of tree peony, a total of 39 flavonoids (5 anthocyanins, 12 flavones, 21 flavonols, and 1 chalcone) have been identified^11,12^. Recently, 56 flavonoids were further characterized from 15 traditional Chinese tree peony cultivars with white, pink, and red color series^13^. In yellow-colored tree peony flowers, 26 flavonoid components have been detected, among which Km, Ap, Lu glucosides, and chalcones were dominant^11^. The main flavonoid components in yellow flowers of *P. lutea* were chalcones, flavones, and flavonols, including tetrahydroxychalcone (THC), isosalipurposide (ISP), Km, Qu, Is, Ch, and Ap^5^. The production of chalconaringenin 2’-*O*-glucoside (Chalcone 2’G) was presumed to be a leading reason for yellow pigmentation in petals of *P. lutea*^14^.

Flavonoid synthesis can be divided into three phases. The first phase includes the transformation of phenylalanine to coumaroyl-CoA, which is shared by many secondary metabolism pathways. The second phase corresponds to the conversion of coumaroyl-CoA to dihydroflavonols, including dihydrokaempferol, dihydroquercetin, and dihydromyricetin (DHM). This process is catalyzed by a battery of enzymes covering chalcone synthase (CHS), chalcone isomerase (CHI), flavanone 3-hydroxylase (F3H), flavonoid 3’-hydroxylase (F3’H), flavonoid 3’5’-hydroxylase, flavone synthase (FNS), and flavonol synthase (FLS). The second phase is critical for yellow coloration due to its accumulation of chalcones, flavones, and flavonols. Subsequently, the third phase is the synthesis of a series of stable anthocyanins catalyzed by dihydroflavonol 4-reductase (DFR), anthocyanidin synthase (ANS), UDP-glucose flavonoid 3-*O*-glucosyltransferase (UFGT), and anthocyanin *O*-methyltransferase^15,16^. These structural genes are regulated by the MBW complex, which contains MYB, bHLH transcription factors (TFs), and WD40 proteins^17^. In the regulation of flavonoid production, MYBs serve as key TFs and bind directly to bHLH regulators or the promoters of structural genes to activate gene expression^18^. As chaperone proteins, WD40 plays a role in stabilizing the MBW complex. In addition, other TFs, such as SPLs, NACs, WRKY, HY5, and ERFs, are also involved in regulating flavonoid biosynthesis^19,20^. In particular, SPLs can disturb the formation of the MBW complex by competing for bHLHs or MYBs, thus inhibiting anthocyanin production^21^. Recently, many flavonoid synthesis-related genes have been identified in tree peony, including the structural genes *PsCHS1*, *Ps-CHI1*, *PsF3H1*, *PsDFR1*, and *PsANS1*, and the TF genes *PlWDR3*, *PlWDR18*, *PlbHLH3*, *PoMYB2*, *PsMYB12*, and *PoSPL1*^2,14,22–28^. However, only a limited number of studies have reported candidate genes for yellow pigmentation in tree peony. For example, transgenic tobacco plants constitutively expressing *Ps-CHI1* exhibit an up to threefold increase in total flavonol and flavone levels, and a significant reduction in anthocyanin content and floral color strength compared to wild-type (WT) controls^24^. High coexpression of *THC2’GT*, *CHI*, and *FNS II* in *P. lutea* flowers guarantees the accumulation of yellow pigments^14^. Notably, no TF gene regulating yellow pigmentation in tree peony has been identified.

Second-generation sequencing (SGS) technology has been widely applied to transcriptomic analysis in tree peony^14,29–31^. Recently, the third-generation sequencing (TGS) platform single-molecule real-time sequencer PacBio RS (Pacific Biosciences of California, USA) has been developed for more advanced RNA sequencing (RNA-seq)^32,33^. Compared to SGS, TGS has longer read lengths, higher consensus accuracy, a lower degree of bias, and simultaneous capability for epigenetic characterization^34^. In turn, the sequence alignment errors of TGS can be algorithmically improved and corrected by SGS reads^35^. Therefore, higher quality transcriptome assemblies can be achieved by combining SGS and TGS technologies.

In this study, we performed metabolomics and full-length transcriptomics analyses of the petal tissues of two tree peony cultivars, “High Noon” (yellow flowers) and “Roufurong” (purple–red flowers), at different flowering stages. Differentially expressed genes (DEGs) and the metabolic profiles of the two cultivars were identified and characterized. Candidate genes responsible for yellow pigmentation were further investigated by quantitative real-time PCR (qRT-PCR) analysis, subcellular localization, overexpression in tobacco, promoter sequence analysis, and dual-luciferase assays. The molecular mechanisms of yellow pigmentation in tree peony flowers were discussed.

## Results

### Assessment of flower color phenotypes

**Fig. 1:**
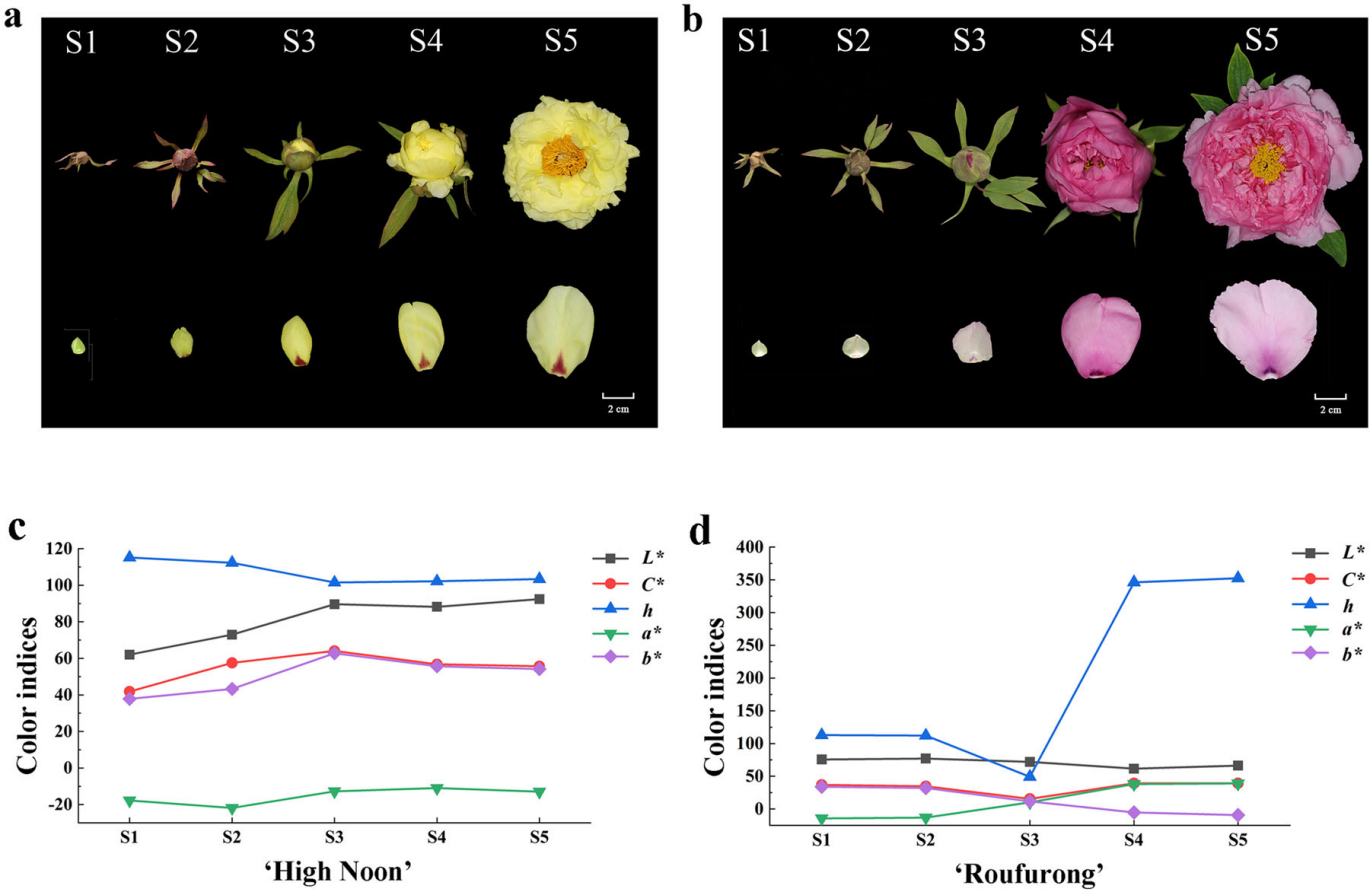
Floral phenotypes and color indices of tree peony cultivars “High Noon” and “Roufurong” at five different blooming stages. **a** Floral phenotype of “High Noon”. **b** Floral phenotype of “Roufurong”. **c** Color indices of “High Noon”. **d** Color indices of “Roufurong”. S1: Stage 1, unpigmented tight bud; S2: Stage 2, slightly pigmented soft bud; S3: Stage 3, initially opened flower; S4: Stage 4, half opened flower; S5: Stage 5, fully opened and pigmented flower with exposed anthers. L* represents the brightness; the color becomes brighter as the value increases. C* represents chroma; the color saturation increases as the value rises. *h* represents the hue angle, defined as follows: 0° (360°) for purple–red, 90° for yellow, 180° for blue–green, and 270° for blue^69^. a* and b* represent the redness and yellowness, respectively

To characterize flower color development in tree peony, the petal tissues of the yellow-flowered cultivar “High Noon” and purple–red-flowered cultivar “Roufurong” were sampled at five developmental stages from early flower buds to full blooms (Fig. 1a, b). The petal color indices of the two cultivars were measured at S1–S5 (Fig. 1c, d). In “High Noon”, the L* (brightness) value increased gradually from S1 to S5, indicating the elevation of petal color brightness. C* (chroma) and b* (yellowness) peaked at S3 and subsequently declined at S4 and S5, demonstrating that S3 was the yellowest stage. In contrast to L*, C*, and b*, *h* (hue angle) decreased from S1 to S3, followed by a slight increase at S4 and S5. For “Roufurong”, no significant change in L* was observed throughout the flowering process. *h* dropped from S1 to S3 and then increased dramatically at S4 and S5. *h*-Values from S3 to S5 were ~0° (360°), consistent with the purple–red color. Similar to *h*, C* in “Roufurong” also decreased from S1 to S3 and increased at the later stages S4 and S5. Consistent with the flower color, b* in the purple–red-colored “Roufurong” was significantly lower than that in the yellow-colored “High Noon” from S1 to S5. Consistently, a* (redness) was much higher in “Roufurong” than in “High Noon”.

### Quantitative analysis of flavonoids

**Fig. 2:**
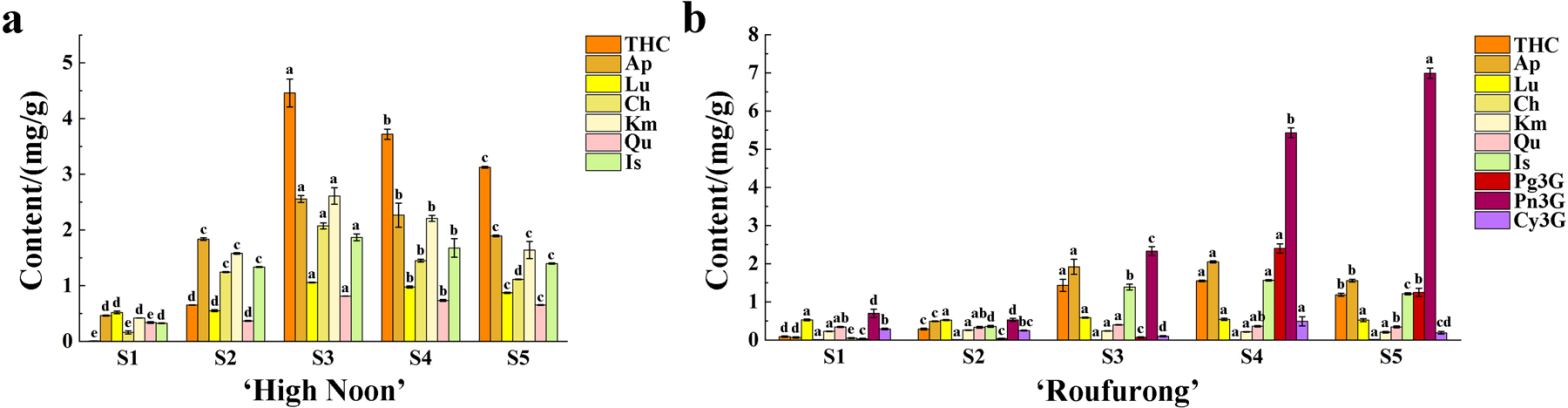
Contents of different flavonoids in petals of “High Noon” and “Roufurong” at five blooming stages. **a** “High Noon”. **b** “Roufurong”. Error bars represent the SE of the mean from three biological replicates. Different letters (**a**–**d**) indicate significant differences (*p* < 0.05) among different flavonoids by Duncan’s test. THC, tetrahydroxychalcone; Ap, apigenin; Lu, luteolin; Ch, chrysoeriol; Km, kaempferol; Qu, quercetin; Is, isorhamnetin; Pg3G, pelargonidin 3-*O*-glucoside; Pn3G, peonidin 3-*O*-glucoside; and Cy3G, cyanidin 3-*O*-glucoside

Significant variations in flavonoid contents were observed between the two tree peony cultivars at each developmental stage. As shown in Fig. 2a, b, the target flavonoids in “High Noon” increased dramatically from S1 to S3 and then dropped slightly at S4 and S5. At the later flowering stages S3–S5, the content of THC was significantly higher than that of other flavonoids. Ap and Km had comparable contents throughout the five flowering stages. In addition, Ch had similar production levels to Is, whereas Lu was similar to Qu. Notably, no anthocyanin was detected in “High Noon” throughout the flowering process. In contrast, three anthocyanins were detected in “Roufurong”. The dominant peonidin 3-*O*-glucoside (Pn3G) increased dramatically as the flower bloomed and reached the maximum level at S5, implying that Pn3G may contribute to purple–red flower pigmentation. Compared to “High Noon”, the THC, Is, and Ap contents showed a quick elevation during the first four periods and a moderate decline at S5. Their variation ranges were relatively smaller than that of pelargonidin 3-O-glucoside (Pg3G). In contrast, the contents of Lu, Km, Qu, cyanidin 3-*O*-glucoside (Cy3G), and Ch did not display a clear developmental pattern, and thus, these components may have no significant influence on the purple–red coloration.

To understand the relationship between flower color and flavonoid components, multiple linear regression (MLR) analysis was performed (Table 1). In “High Noon”, L* and b* were positively correlated with THC levels, indicating that the increase in the THC content led to the accumulation of yellow pigments. In contrast, the *h*-value was negatively correlated with THC levels. Ch had a positive effect on C* but a negative effect on a*. Therefore, THC and Ch may be associated with the production of yellow flowers. In “Roufurong”, Pg3G had a negative impact on L* but a positive impact on *h*, suggesting that a high content of Pg3G may lead to a low brightness of the petals. In addition, C* was negatively correlated with the Lu content, which may affect petal saturation as a copigment. Moreover, a* was found to be positively and negatively associated with Pn3G and Ch, respectively, whereas b* had a negative correlation with Pn3G and Ap. These results indicated that a high content of Pn3G and a low content of Ch were critical for the purple–red color of petals. Considering that THC and Ch displayed the highest accumulation levels at S3 in “High Noon”, and C* and b* also peaked at S3, stage S3 was further selected for subsequent transcriptome analyses to identify the candidate genes for yellow pigmentation.

**Table 1:** Multiple linear regression equations of two tree peony cultivars, “High Noon” and “Roufurong”

Cultivars	Equations
“High Noon”	L* = 66.173 + 6.214 THC (*R*^2^ = 0.867, *P* < 0.05)
	C* = 41.198 + 11.574 Ch (*R*^2^ = 0.960, *P* < 0.01)
	*h* = 114.597 − 3.215 THC (*R*^2^ = 0.979, *P* < 0.01)
	a* = −30.806 + 26.860 Lu − 4.801 Ch (*R*^2^ = 0.993, *P* < 0.05)
	b* = 38.558 + 5.095 THC (*R*^2^ = 0.980, *P* < 0.01)
“Roufurong”	L* = 74.970 − 5.889 Pg3G (*R*^2^ = 0.908, *P* < 0.05)
	C* = 214.880 − 335.919 Lu (*R*^2^ = 0.827, *P* < 0.05)
	*h* = −164.139 + 122.546 Pg3G + 22840.617 Ch (*R*^2^ = 0.999, *P* < 0.01)
	a* = 7.308 + 9.695 Pn3G − 2261.699 Ch (*R*^2^ = 0.997, *P* < 0.05)
	b* = 38.353 − 5.126 Pn3G − 7.639 Ap (*R*^2^ = 0.999, *P* < 0.01)

a* redness, *Ap* apigenin, b* yellowness, C* chroma, *Ch* chrysoeriol, *Cy3G* cyanidin 3-*O*-glucoside, *h* hue angle, *Is* isorhamnetin, *Km* kaempferol, L* brightness, *Lu* luteolin, *Pg3G* pelargonidin 3-*O*-glucoside, *Pn3G* peonidin 3-*O*-glucoside, *Qu* quercetin, *THC* tetrahydroxychalcone

### Overview of transcriptome sequencing

To obtain the reference transcriptome assemblies for tree peony, ten petal samples at S1–S5 from “High Noon” and “Roufurong” were pooled for TGS sequencing. A total of 473,062 error-corrected reads of insert (ROIs) were obtained, with an average read length of 1,883 bp (Supplementary Table 1). Furthermore, 76.1% of the full-length ROIs were considered full-length nonchimeric (FLNC) reads (Supplementary Table 2) and 117,680 high-quality isoforms were obtained after filtering the low-quality consensus sequences (Supplementary Table 3). Transcriptome analyses of petal tissues of the two cultivars at S3 were carried out by the SGS approach with three biological replicates. Six libraries covered 45.88 Gb clean read data with high Q30 (>94%) (Supplementary Table 4). In total, 56,610 open reading frames (ORFs) were mapped to the assembly, of which 41,138 displayed full-length sequences. To estimate the functions of assembled sequences, 56,974 transcripts were annotated by searching against different databases (Supplementary Table 5). The transcripts from tree peony shared the highest similarity with those from *Vitis vinifera* (27.40%) and more transcripts fell under biological process, of which metabolic process, cellular process, and single-organism process were mainly enriched with transcripts (Supplementary Fig. 1a, b). Furthermore, 27,265 transcripts were mapped to 125 metabolic pathways (Supplementary Table 6). Specifically, 115, 12, and 30 transcripts were found in the flavonoid biosynthesis pathway (ko00941), anthocyanin biosynthesis pathway (ko00942), and flavone and flavonol biosynthesis pathway (ko00944), respectively (Supplementary Table 6).

### DEGs involved in flavonoid biosynthesis

**Fig. 3:**
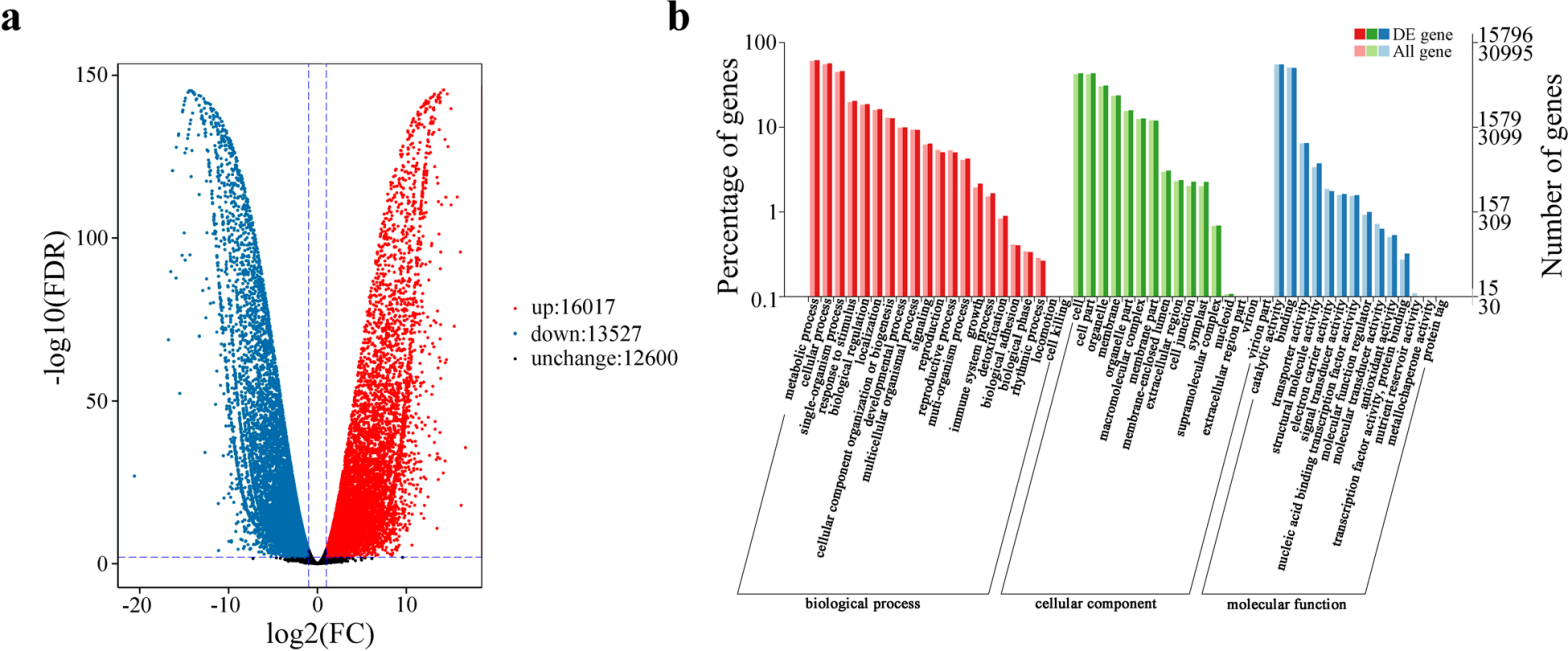
Analysis of differentially expressed genes (DEGs) in “Roufurong” and “High Noon” libraries. **a** Volcano plot of DEGs. **b** GO enrichment analysis of DEGs

**Fig. 4:**
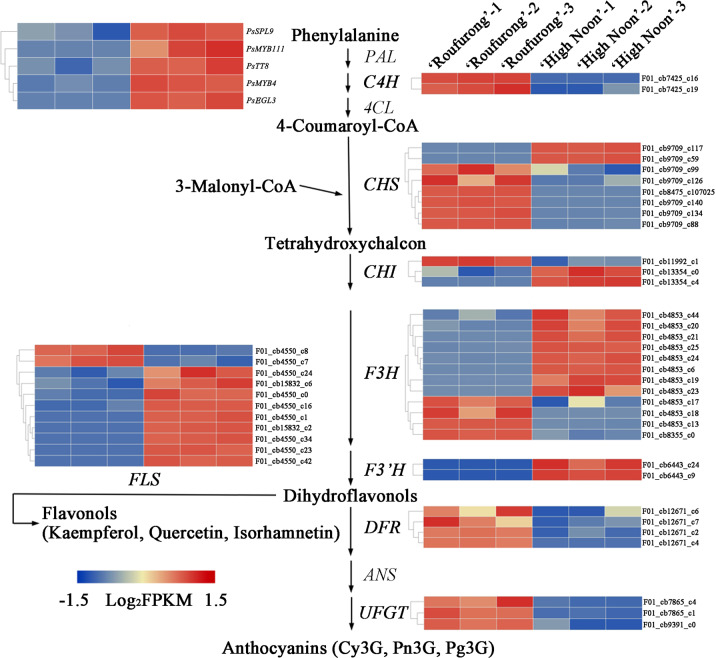
Putative DEGs in the flavonoid biosynthesis pathway and their expression levels at S3 in “High Noon” and “Roufurong”. The expression pattern of each gene is shown in a heatmap beside each step. Blue indicates low expression and red indicates high expression

To identify the candidate genes for yellow pigmentation, DEGs between “High Noon” and “Roufurong” at S3 were identified. There were 16,017 upregulated DEGs and 13,527 downregulated DEGs in “High Noon” compared with “Roufurong” (Fig. 3a). Gene Ontology (GO) enrichment analysis revealed that 15,796 DEGs were distributed into 51 terms comprising 21 biological processes, 16 cellular components, and 14 molecular functions. Among the largest category, biological process, the DEGs were significantly enriched in “metabolic process”, “cellular process”, and “single-organism process” (Fig. 3b). DEGs involved in flavonoid biosynthesis were screened and contained 45 structural genes (2 *PsC4H*s, 8 *P*s*CHS*s, 3 *P*s*CHI*s, 12 *PsF3H*s, 2 *PsF3’H*s, 11 *PsFLS*s, 4 *PsDFR*s, and 3 *PsUFGT*s) and 5 TF genes (*PsMYB4* (F01_cb7851_c8), *PsMYB111* (F01_cb8732_c11), *PsTT8* (F01_cb3692_c7), *PsEGL3* (F01_cb8475_c28561), and *PsSPL9* (F01_cb9156_c0)). The expression profiles of these DEGs related to flavonoid biosynthesis are displayed in Fig. 4. Twelve of these genes were randomly selected for qRT-PCR to validate the transcriptome data (Supplementary Fig. 2). Their expression levels were consistent with the RNA-seq data results.

As shown in Fig. 4, structural genes at the second stage of flavonoid biosynthesis generally presented higher expression levels in “High Noon” than in “Roufurong”. These genes included two *PsCHS*s (F01_cb9709_c117 and F01_cb9709_c59) and two *PsCHI*s (F01_cb13354_c0 and F01_cb13354_c4). Based on gene annotation, *PsCHS* functions in the synthesis of THCs, whereas *PsCHI* plays a critical role in the production of flavanones, which contribute to yellow coloration. In addition, eight putative *PsF3H*s (F01_cb4853_c44, F01_cb4853_c20, F01_cb4853_c21, F01_cb4853_c25, F01_cb4853_c24, F01_cb4853_c6, F01_cb4853_c19, and F01_cb4853_c23), two *PsF3’H*s (F01_cb6443_c24 and F01_cb6443_c9), and nine *PsFLS*s (F01_cb4550_c24, F01_cb15832_c6, F01_cb4550_c0, F01_cb4550_c16, F01_cb4550_c1, F01_cb15832_c2, F01_cb4550_c34, F01_cb4550_c23, and F01_cb4550_c42) also exhibited higher transcription in “High Noon”. Of these genes, *PsFLS* can divert the flavonoid pathway in favor of flavonol production. These expression variations between “High Noon” and “Roufurong” were consistent with the observed higher levels of THC, flavones, and flavonols in “High Noon”. In contrast to the structural genes at the second stage, genes at the third stage of flavonoid biosynthesis, including four *PsDFRs* (F01_cb12671_c6, F01_cb12671_c7, F01_cb12671_c2, and F01_cb12671_c4) and three *PsUFGTs* (F01_cb7865_c4, F01_cb7865_c1, and F01_cb9391_c0), were significantly downregulated in “High Noon”. *PsDFR* and *PsUFGT* are responsible for the biosynthesis of anthocyanins such as Cy3G, Pn3G, and Pg3G. Therefore, the downregulation of *PsDFR*s and *PsUFGT*s in “High Noon” may explain the lack of anthocyanin production in this yellow-flowered cultivar.

TF genes regulating flavonoid biosynthesis were also analyzed with the transcriptomes of “High Noon” and “Roufurong”. As shown in Fig. 4, all of these identified TFs were upregulated in the yellow-flowered “High Noon.” The interaction between MYB and bHLH TFs plays a key role in the regulation of structural genes in the flavonoid biosynthetic pathway^17^. Candidate MYB proteins related to flavonoid biosynthesis were identified by phylogenetic analyses with MYBs from *Arabidopsis*. The results showed that PsMYB4 belonged to subgroup 4 (S4) of *Arabidopsis* (Supplementary Fig. 3a). S4 MYBs in *Arabidopsis* directly interact with AtbHLHs (TT8, GL3, and EGL3) and repress the transcriptional activities of MBW complexes, thereby leading to the inhibition of anthocyanin and phenylpropanoid syntheses^36^. PsMYB111 was clustered to S7 (Supplementary Fig. 3a), which was shown to regulate flavonol biosynthesis in *Arabidopsis*^37^. Sequence analysis revealed that PsMYB4 and PsMYB111 were identified as typical R2R3MYB proteins (Supplementary Fig. 3b, c), and PsMYB4 contained the conserved sequence [D/E]LX_2_[K/R]X_3_LX_6_LX_3_R, which interacts with bHLH proteins. Notably, although PsMYB111 does not interact with bHLHs, the SG7 motif [K/R][R/X][R/K]XGRT[S/X][R/G]XX[M/X]K and SG7-2 motif [W/X][L/X]LS, which are specific to flavonol biosynthesis regulators^38,39^, were found at the C terminus (Supplementary Fig. 3c). In the case of bHLHs, PsTT8 and PsEGL3 were identified in the S5 subgroup (Supplementary Fig. 4a) and were related to flavonoid biosynthesis. Both PsTT8 and PsEGL3 contained an N-terminal MYB-interacting region (MIR), a bHLH domain, and a putative ACT-like domain at the C terminus (Supplementary Fig. 4b). MIR suggests that PsTT8 and PsEGL3 may interact with MYB proteins, and the putative ACT-like domain has been shown to play a vital role in the dimerization of bHLHs^40^. In addition, phylogenetic analysis suggested that PsSPL9 containing a typical squamosa promoter-binding protein domain was clustered together with AtSPL9 and AtSPL15 (Supplementary Fig. 5a, b), of which AtSPL9 has been reported to inhibit the accumulation of anthocyanin by destroying the stability of the MBW complex in *Arabidopsis*^21^.

### Expression patterns of candidate DEGs related to flavonoid biosynthesis

**Fig. 5:**
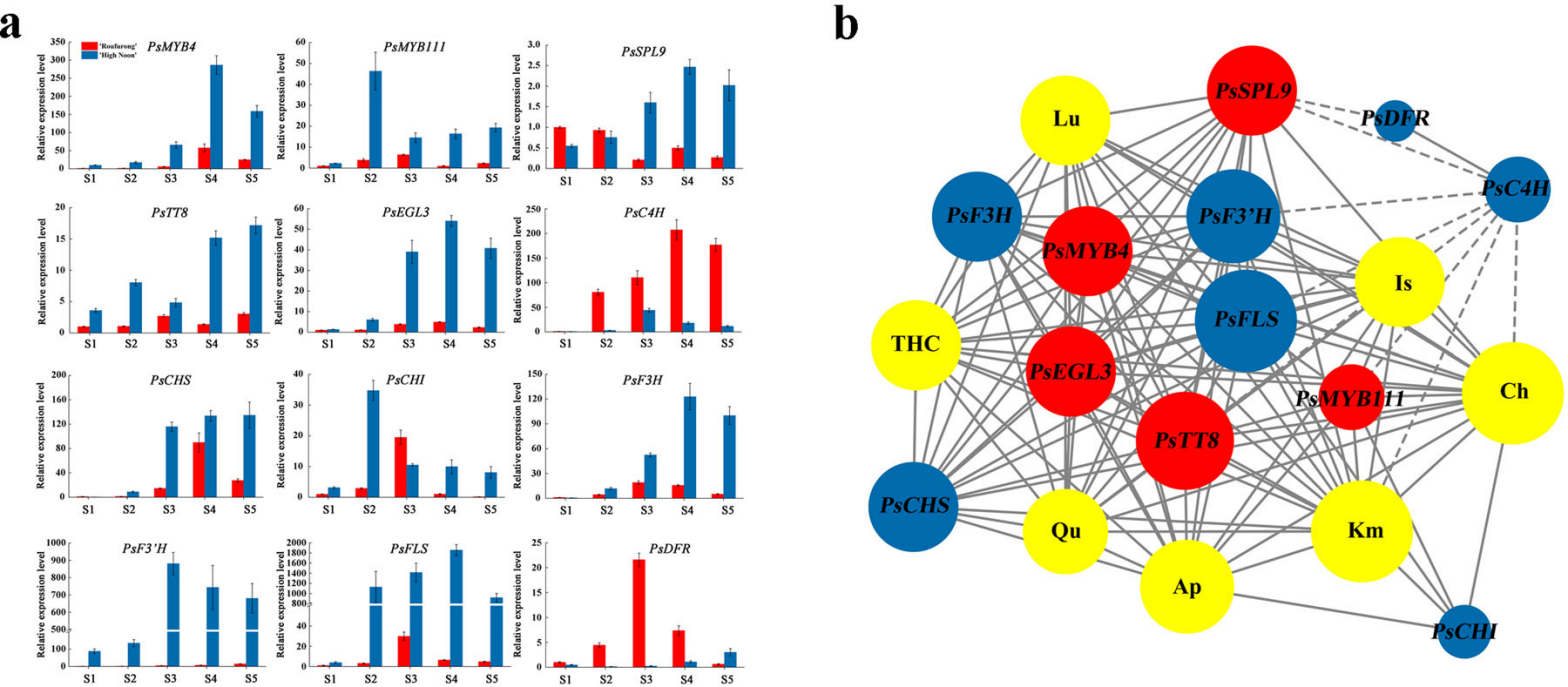
Expression analysis of selected transcription factor (TF) genes and structural genes at five blooming stages in the petals of “High Noon” and “Roufurong”. **a** Expression patterns of selected genes at five blooming stages in the petals of “High Noon” and “Roufurong”. **b** An interaction network between selected genes and flavonoids. Red represents TF genes, blue represents structural genes, and yellow represents flavonoids. The solid line represents a positive correlation and the dashed line represents a negative correlation. A larger circle and more nodes represent a stronger correlation

qRT-PCR analyses were conducted to investigate the expression patterns of structural genes *PsC4H* (F01_cb7425_c19), *PsCHS* (F01_cb9709_c126), *PsCHI* (F01_cb13354_c0), *PsF3H* (F01_cb4853_c44), *PsF3’H* (F01_cb6443_c9), *PsFLS* (F01_cb4550_c24), and *PsDFR* (F01_cb12671_c6), as well as TF genes *PsMYB4*, *PsMYB111*, *PsTT8*, *PsEGL3*, and *PsSPL9* at five flowering stages, S1–S5, in “High Noon” and “Roufurong” petals (Fig. 5a). For structural genes, only *PsC4H* and *PsDFR* were expressed at higher levels in “Roufurong”, which was consistent with the accumulation of anthocyanins (Fig. 2b). In contrast, the other selected structural genes, *PsCHS*, *PsCHI*, *PsF3H*, *PsF3’H*, and *PsFLS*, all displayed higher expression in “High Noon”. Furthermore, the expression patterns of these genes from S1 to S5 were generally consistent with the previously determined flavonoid accumulation pattern (Fig. 2a). *PsCHS* transcription at S3 was greatly upregulated and high expression was maintained at both S4 and S5, whereas the expression of *PsCHI* peaked at S2 and then significantly decreased at S3, S4, and S5. The high expression of *PsCHS* and the low expression of *PsCHI* led to the accumulation of THC from S3 to S5. In addition, the high expression of *PsF3H*, *PsF3’H*, and *PsFLS* at S3, S4, and S5 might have guided the metabolic flow in favor of the production of flavonols such as Km, Qu, and Is. Notably, the expression of *PsFLS* and *PsDFR* in “High Noon” was negatively related, suggesting a potential competitive mechanism between these two genes. In terms of TF genes, *PsMYB4*, *PsEGL3*, and *PsSPL9* in “High Noon” had a consistent expression pattern that first increased and then declined at later stages. This pattern was in contrast to the expression pattern of *PsDFR*. The expression patterns of *PsMYB111* and *PsTT8* were similar in “High Noon”, which peaked at S2, dropped dramatically at S3, and then increased slightly at S4 and S5. This expression pattern exactly matched that for *PsCHI* and *PsFLS*, leading to the assumption that PsMYB111 may directly regulate the transcription of these two structural genes.

Based on the gene expression and flavonoid accumulation profiles, an interaction network between TF genes and structural genes encoding flavonoids was developed (Fig. 5b). The results showed that *PsSPL9* was negatively correlated with *PsDFR* and *PsC4H*. A strong positive correlation was observed between *PsMYB4* and *PsEGL3*, as well as between *PsMYB111* and *PsTT8*. These four TF genes acted as hub genes in the whole interaction network. The interaction network also showed that *PsFLS* was closely related to *PsMYB111* and *PsTT8*, and directly associated with Km, Qu, and Is. Nevertheless, PsMYB111 has been identified as devoid of bHLH interaction sites; thus, we hypothesized that PsMYB111 might affect the production of flavonols by regulating the expression of *PsFLS* alone. To validate this hypothesis, *PsMYB111* was selected for subsequent functional analysis.

### Subcellular localization analysis of *PsMYB111*

**Fig. 6:**
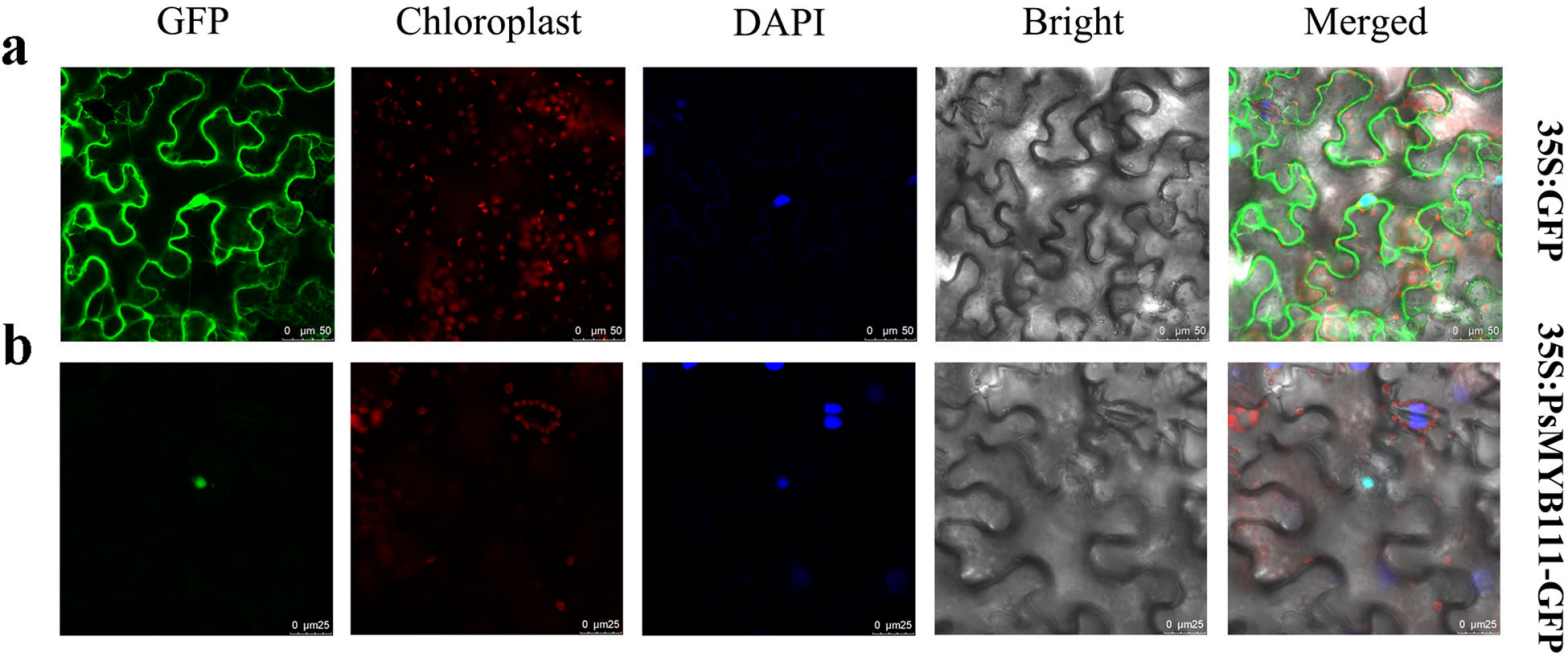
Subcellular localization analysis of the PsMYB111 fusion protein in tobacco leaves. **a** Control vector (pCAMBIA1302-GFP) expressed in epidermal cells of tobacco leaves. **b** Recombinant vector (PsMYB111-GFP) expressed in epidermal cells of tobacco leaves. GFP, GFP fluorescence; Chloroplast, chloroplast fluorescence; DAPI, DAPI fluorescence; Bright, bright field; and Merged, superposition of bright field and fluorescence. Bars, 25 and 50 µm

To explore the subcellular localization of PsMYB111, a plasmid containing *PsMYB111* fused to green fluorescent protein (GFP) was constructed and transiently transformed into *Nicotiana benthamiana* leaves. The subcellular localization of the protein was observed after 72 h. Fluorescence of the fusion protein PsMYB111-GFP was detected specifically in the nucleus (Fig. 6a, b), supporting the role of PsMYB111 as a TF involved in regulating flavonol biosynthesis.

### Overexpression of *PsMYB111* in tobacco

**Fig. 7:**
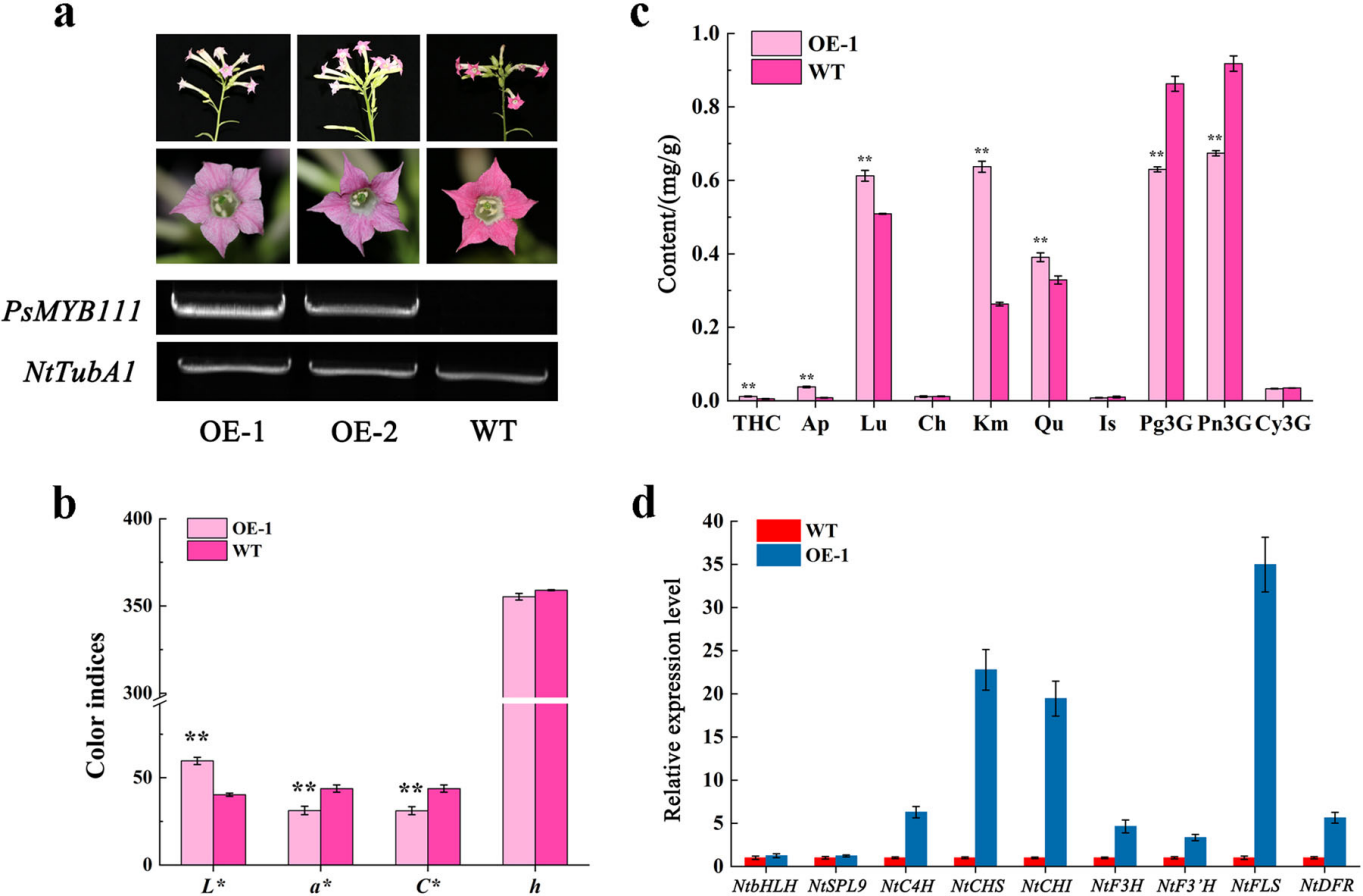
Overexpression of *PsMYB111* increases the accumulation of flavonols and reduces the accumulation of anthocyanins in tobacco. **a** Flowers from wild type (WT) and transgenic tobacco plants, and their corresponding *PsMYB111* transcription levels. *NtTubA1* was used as an endogenous control. **b** Color indices of petals from WT and transgenic tobacco plants. **c** Flavonoid contents in petals of WT and transgenic tobacco plants. **d** Expression patterns of endogenous TF genes and flavonoid biosynthetic genes in the petals of WT and transgenic tobacco plants. Three independent experiments were performed for each sample. The data are shown as the means ± SDs. **p < 0.01

To further characterize the function of *PsMYB111* in flavonoid biosynthesis, we constructed transgenic tobacco lines overexpressing *PsMYB111* (*PsMYB111* OE). Two T_2_ lines (OE-1 and OE-2) were obtained. The flower color of OE-1 and OE-2 was light pink, in contrast to the rosy red color of the WT (Fig. 7a). OE-1 displayed a higher *PsMYB111* transcript level than OE-2 and was thus selected for further study (Fig. 7a). The color indices L*, a*, C*, and *h* of tobacco petals were measured. The results showed that the L*-value of OE-1 was significantly higher than that of WT, whereas the values of a* and C* decreased significantly (*P* < 0.01) (Fig. 7b). Correspondingly, the contents of THC, Ap, Lu, Km, and Qu also increased significantly in OE-1 compared to WT, whereas the contents of Pg3G and Pn3G decreased significantly (*P* < 0.01) (Fig. 7c). These results were consistent with the color phenotype of the transgenic tobacco.

To investigate whether PsMYB111 can regulate the transcription of flavonoid biosynthetic structural genes, we analyzed the expression of selected structural genes in OE-1 petals using qRT-PCR (Fig. 7d). The results showed that the transcription of *NtCHS*, *NtCHI*, and *NtFLS* increased significantly compared to WT, whereas *NtC4H*, *NtF3H*, *NtF3’H*, and *NtDFR* were only mildly upregulated. The upregulation of *NtFLS* was the highest among the analyzed structural genes. In addition, *NtbHLH* and *NtSPL9* exhibited similar expression abundances to WT. Taken together, PsMYB111 may regulate the expression of structural genes alone rather than in a complex and may enhance the accumulation of flavonols such as Qu and Km.

### Regulation of *PsCHS* and *PsFLS* promoters by PsMYB111

**Fig. 8:**
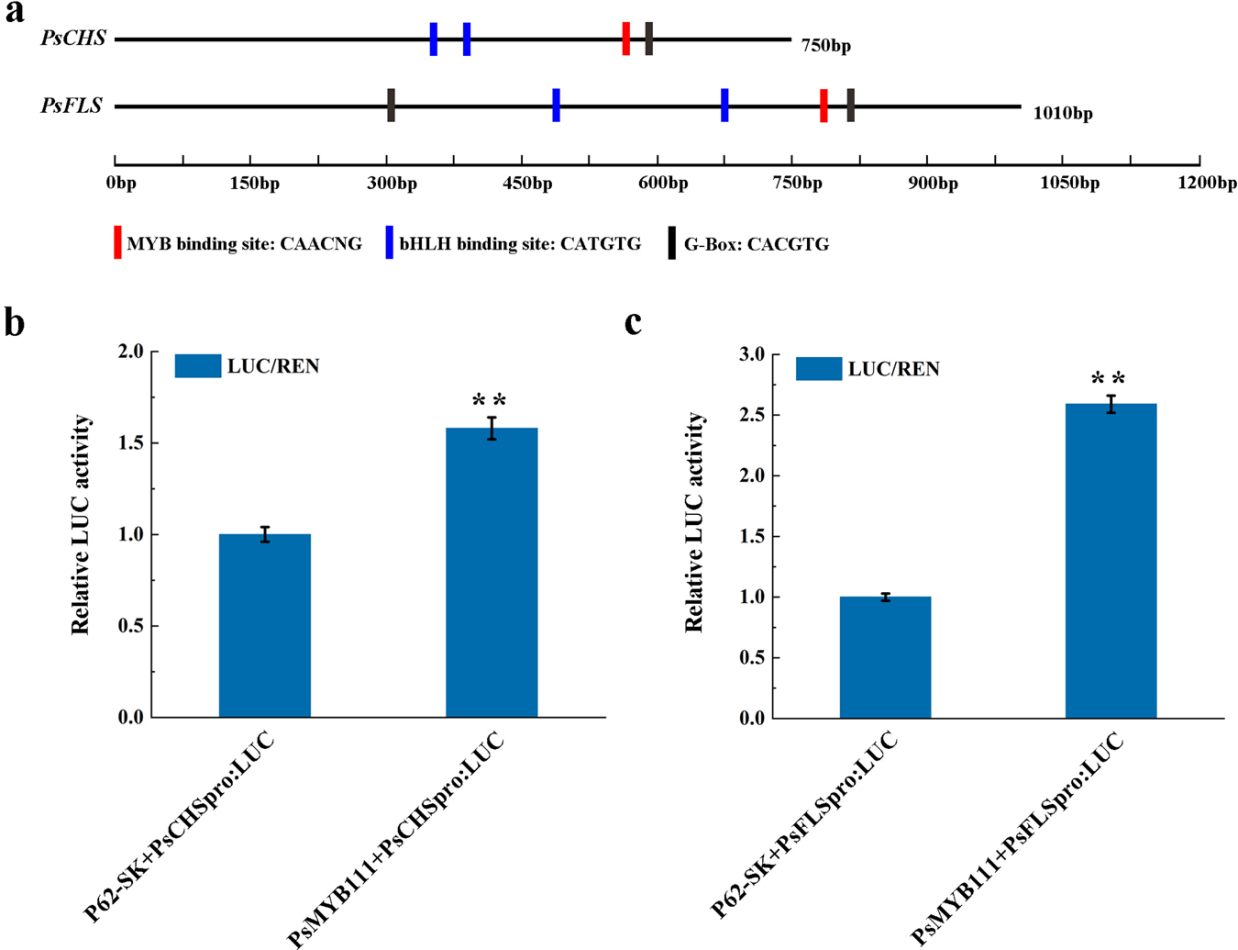
Transcriptional activity analysis of PsMYB111 against the promoters of *PsCHS* and *PsFLS* of tree peony. **a** Schematic overview of *PsCHS* and *PsFL*S promoters. **b** Dual-luciferase assays in tobacco leaves. Transformed protoplasts including only a promoter-LUC reporter construct without an effector were used as controls. The data represent the means ± SDs of three replicates from three independent experiments. **p < 0.01

Transgenic experiments in tobacco leaves indicated that PsMYB111 can directly regulate *NtCHS* and *NtFLS*. To verify its regulatory role on *PsCHS* and *PsFLS*, we sequenced the promoter sequences of *PsCHS* (750 bp) and *PsFLS* (1,010 bp), and analyzed their *cis*-acting elements (Fig. 8a). Many *cis*-acting elements related to MYB and bHLH TFs were identified, including the MYB-binding site (5′-CAACNG-3′), bHLH-binding site (5′-CATGTG-3′), and G-box (5′-CACGTG-3′) (Fig. 8a), supporting their potential interaction with PsMYB111.

To further confirm this hypothesis, a dual-luciferase assay was performed. The pGreenII62-SK vector carrying PsMYB111 served as an effector and the pGreenII0800 LUC vector carrying the promoters of *PsCHS* and *PsFLS* served as reporters. After coinfiltration of the TF and promoters in *N. benthamiana* leaves, the LUC/REN ratios were detected. As shown in Fig. 8b, c, significantly higher LUC/REN ratios were observed for both the PsMYB111-*PsCHS* and PsMYB111-*PsFLS* constructs compared with the corresponding controls, suggesting that PsMYB111 can activate the promoters of both *PsCHS* and *PsFLS*. The activation intensity of PsMYB111 on *PsFLS* (2.59-fold) was stronger than that of *PsCHS* (1.58-fold), indicating that PsMYB111 may preferentially regulate the transcription of *PsFLS*.

## Discussion

Pure yellow flowers in tree peony are extremely rare. The molecular mechanisms of yellow pigmentation in this species remain unclear. Here we selected the cultivar “High Noon” with yellow flowers as the research material, with the purple–red-flowered cultivar “Roufurong” as a control. The floral color phenotypes and flavonoid profiles of “High Noon” petals were characterized, together with combined full-length and comparative transcriptome analyses. Candidate genes underlying yellow pigmentation were further validated by functional analyses.

Flavonoids play a key role in the coloration of different plant organs. In this study, we showed that the contents of THC, flavones (Ap, Lu, and Ch), and flavonols (Km, Qu, and Is) in the flowers of “High Noon” were markedly higher than in those of “Roufurong”. No anthocyanin was detected in the yellow petal tissues of “High Noon”. Similar observations have been found for yellow-flowered *P. lutea* from Yunnan, which also contains abundant THC, ISP, Km, Qu, Is, Ch, and Ap^5^. Among these compounds, THC and Ch were the main pigments causing yellow color. The high amount of Chalcone 2’G has been speculated to be an important reason for the yellow-flowered phenotype in *P. lutea*^14^. In “Roufurong”, the accumulation of the dominant anthocyanin Pn3G is the most likely reason for its purple–red-colored petals. It has been reported that reductions in anthocyanins increased the L*-value and decreased the a*-value, causing flower colors of two herbaceous peony cultivars “Coral Sunset” and “Pink Hawaiian Coral”, to change from coral to pink and then to yellow^41^. In addition, the sharp decrease in anthocyanins during flowering of “Jinyi Hualian” and “Xiaguang” elicited colors from red to orange and then to yellow^42^. Therefore, the high contents of THC, flavones, and flavonols without anthocyanins may be essential for the yellow pigmentation of “High Noon” flowers.

In the flavonoid biosynthetic pathway, CHS catalyzes the synthesis of a key intermediate THC, which can be further isomerized by CHI, leading to the production of flavones and flavonols^14^. It was previously reported that a low expression level of *PlCHI* in the *Paeonia lactiflora* cultivar “Huangjinlun” induced chalcone accumulation and produced a yellow color^43^. In the present study, we assume that the high expression of *PsCHS* and low expression of *PsCHI* in “High Noon” during S3–S5 contributed to the large accumulation of THC, which also resulted in yellow coloring. In addition, the high expression of *PsF3H*, *PsF3’H*, and *PsFLS* from S3 to S5 in “High Noon” promoted metabolic flow to the synthesis of flavonols (Km, Qu, and Is). In white *Muscari armeniacum* flowers, when *FLS* was upregulated, the substrates used for cyanidin synthesis were then available for the synthesis of kaempferol^44^. The downregulation of *PsDFR* and *PsUFGT* is consistent with the lack of anthocyanin accumulation in “High Noon”. Similarly, observations have been made in *P. lactiflora*, in which the lower expression levels of *PlDFR*, *PlANS*, *Pl3GT*, and *Pl5GT* in the inner petals inhibited anthocyanin production, resulting in yellow pigment formation^45^. Notably, the substrate competition mechanism between FLS and DFR may cause variations in anthocyanin and flavonol synthesis, as FLS strengthens dihydroflavonol flux toward flavonols and finally limits anthocyanin accumulation^46^. In the present study, we also observed competition between FLS and DFR, which may have also contributed to the yellow-flowered phenotype in “High Noon”. The upregulated expression of *FLS* transcripts in “High Noon” may consume dihydroflavonols as a substrate, thereby increasing the accumulation of flavonols and reducing the accumulation of anthocyanins at the DFR branch. Similar findings have been revealed in *Paeonia ostii*, which exhibited upregulated expression of *PoFLS4* and a transition of dihydroflavonols into flavonols in nearly white flowers^28^. The competition between FLS and DFR in *M. armeniacum* for DHM might inhibit the synthesis of delphinidin, thereby altering the ratio of flavonol to anthocyanin and furthering the elimination of blue pigmentation^44^. The latest research on *Brassica oleracea* L. *italica* also showed that the difference in kaempferol accumulation was likely caused by the expression level of *FLS*^47^. In contrast, the increased transcription of *PlDFR* in pigmented flowers of *Pleione limprichtii* was accompanied by a decrease in *PlFLS* transcription, causing increased production of anthocyanins^48^. In addition, the competition mechanism between FLS and DFR has also been reported to underlie the lack of anthocyanins in white-fruited Chinese bayberry^27^.

MYB and bHLH are critical TFs that regulate flavonoid biosynthesis in plants. In this study, we identified several differentially expressed MYB and bHLH TFs, each of which is related to flavonoid production in tree peony. Of these, PsMYB4 was identified as a negative flavonoid regulator with bHLH interaction sites, implying that they might form a complex to negatively regulate downstream structural genes. In *Arabidopsis*, the triple mutant *Atmyb4/7/32* displayed elevated anthocyanin and phenylpropanoid accumulation compared to the WT plants, which is consistent with our results^36^. We also observed that *PsDFR* and *PsC4H* were obviously negatively correlated with *PsSPL9*. The high expression of *PsSPL*9 and low expression of *PsDFR* may inhibit anthocyanin production in “High Noon”. Similarly, the AtSPL9 TF in *Arabidopsis* was also proven to inhibit the expression of anthocyanin biosynthetic genes, particularly *DFRs*^21^. In addition, yellow coloration in the floral tissues of *P. lactiflora* is possibly under the regulation of miR156e-3p-targeted *SPL1* by suppressing *PlPAL*, *PlFLS*, *PlDFR*, *PlANS*, *Pl3GT*, and *Pl5GT*^45,49^. For *PsMYB111* and *PsTT8*, although they displayed a consistent expression pattern (Fig. 5a), PsMYB111 was most likely free of bHLH interactions. Based on homology clustering, PsMYB111 belonged to the S7 MYBs of *Arabidopsis* (Supplementary Fig. 3a), which have been proven to positively regulate early biosynthetic genes in the flavonoid pathway, such as *CHS*s, *CHI*s, *F3H*s, *F3’H*s, and *FLS*s^37^. AtMYB11, AtMYB12, and AtMYB111 in S7 of *Arabidopsis* function independently of bHLHs^50^. In *V. vinifera*, a similar regulatory model wherein VvMYBF1 was a specific activator of *VvFLS1* and resulted in flavonol accumulation has also been proposed^38^. In this study, we observed a clear association between *PsMYB111* and *PsFLS* transcription. PsMYB111 promotes the accumulation of flavonols by individually regulating *PsFLS*. In addition, PsMYB4 and PsEGL3 may form a complex to negatively regulate some structural genes, whereas PsSPL9 may negatively regulate *PsDFR* alone and inhibit the generation of anthocyanins.

Subcellular localization analysis revealed that PsMYB111 was localized to the nucleus (Fig. 6a, b), indicating that PsMYB111 might function as a TF in the nucleus. Overexpression of *PsMYB111* in tobacco caused its floral color to change from rosy red to light pink. The contents of flavonols such as Km and Qu in transgenic tobacco lines were significantly increased, whereas the contents of anthocyanins such as Pg3G and Pn3G were significantly decreased, thereby confirming its function in the yellow pigmentation of tree peony flowers. In *Gerbera hybrida*, *GhMYB1a* overexpression also led to a significant increase in Km-type flavonol production and a significant decrease in anthocyanin production^39^. In our *PsMYB111*-overexpressing line, we observed an inverse correlation between flavonol and anthocyanin contents, which reflects the competition between these two metabolic fluxes. Heterologous expression of S7 MYBs can regulate the expression of flavonoid biosynthetic genes, especially by upregulating flavonol pathway genes, causing intensive flavonol synthesis and the inhibition of anthocyanin generation^51,52^. Consistent with the increased flavonol levels, overexpression of *PsMYB111* in tobacco led to the increased expression of *NtCHS*, *NtCHI*, and *NtFLS* (Fig. 7d). It has been reported that flavonol genes (*PAL*, *CHS*, *CHI*, *F3H*, and *FLS*) could be generally upregulated in transgenic tobacco overexpressing flavonol-specific MYB TF genes^53,54^. The overexpression of *GtMYBP3* and *GtMYBP4* identified in *Gentiana triflora* promoted the expression of flavonol biosynthesis genes in tobacco and *Arabidopsis*^51^. In addition, the expression of *NtCHS*, *NtF3H*, and *NtFLS* was strongly upregulated in *GhMYB1a*-overexpressing transgenic tobacco lines and GhMYB1a significantly activated the promoters of *NtCHS* and *NtFL*S over *GhDFR* and *GhMYB10* in gerbera^39^. Similarly, we showed that PsMYB111 had a significant activation effect on *PsCHS* and *PsFLS* promoters, particularly *PsFLS* (Fig. 8b, c).

Taken together, our study showed that PsMYB111 may influence the accumulation of flavonols by directly regulating the expression of *PsFLS* and reducing the flux to anthocyanin synthesis, thus ultimately contributing to the formation of yellow flowers in tree peony. The present study not only provides new insights into the regulatory mechanism of flavonol biosynthesis in tree peony but also identifies a potential MYB regulator that may be applied to the molecular breeding of yellow flower tree peony cultivars. In addition, PsMYB4 may interact with PsEGL3 to reduce the synthesis of anthocyanins by negatively regulating some structural genes, whereas PsSPL9 may inhibit the accumulation of anthocyanins by negatively regulating *PsDFR* alone. The functions of these candidate regulators require further study.

## Materials and methods

### Plant materials

*P. suffruticosa* plants were grown in the Tree Peony Garden of Northwest A&F University, Shaanxi Province, China (34°26′ N, 108°07′ E). Two cultivars, “High Noon” with pure yellow flowers and “Roufurong” with purple–red flowers, were used as the experimental materials. All selected plants were grown in fields with adequate light and moisture (Fig. 1). Petal samples were collected from March to April 2018 at five flowering stages, which were characterized by Zhou et al.^5^. The color-related values (L*, a*, b*, C*, and *h*) of fresh petals at these five stages were measured by a tristimulus color meter (CR-400, Konica Minolta, Osaka, Japan). The materials for other tests were quickly frozen in liquid nitrogen and then stored at −80 °C until further use. Tobacco plants (*N. benthamiana and Nicotiana tabacum)* were cultivated in an incubator at 25 °C with a 16/8 h day/night photoperiod. The color-related values (L*, a*, C*, and *h*) of *N. tabacum* petals were measured according to standard methods for tree peony.

### Measurement of flavonoids

Flavonoids were detected using a previously reported high-performance liquid chromatography (HPLC) method with some modifications^11^. Frozen petal samples of tree peony and tobacco were promptly ground to a powder in liquid nitrogen with mortars and pestles. Approximately 300 mg of tree peony petal powder and 100 mg of tobacco petal powder were divided into two parts and dissolved in 6 ml (for tree peony)/2 ml (for tobacco) of methanol-hydrochloric acid (99 : 1, V/V) solution for anthocyanin detection and 6 ml (for tree peony)/2 ml (for tobacco) of methanol solution for the detection of other flavonoids. Next, the samples were leached in the dark at 4 °C for 24 h and shaken and mixed once every 6 h. After 30 min of ultrasound-assisted extraction, the samples were centrifuged at 10,000 r.p.m. for 10 min to collect the liquid supernatants. Finally, each supernatant was filtered through a 0.22 μm nylon microporous membrane. Ten microliters of pure supernatant was quantified by HPLC (LC-2030C 3D, Shimadzu, Kyoto, Japan) equipped with a diode array detector. A 4.6 × 250 mm C18 column (Shimadzu, Kyoto, Japan) was used. Eluent A was a 0.04% formic acid aqueous solution and eluent B was acetonitrile. The following gradient elution conditions were used: 5% B at 0 min, 40% B at 40 min, 100% B at 45 min, 100% B at 55 min, and 5% B at 60 min. The flow rate was 0.5 ml/min at a 40 °C column temperature. Standards of THC, Ap, Lu, Ch, Km, Qu, Is, Cy3G, Pn3G, and Pg3G were purchased from Shanghai Yuanye Bio-Technology Co., Ltd (Shanghai, China). Mean values and SDs were calculated from three independent biological replicates.

Furthermore, MLR analysis was conducted by SPSS (version 23.0) to explore the relationship between flower colors and flavonoid components. The contents of THC, Ap, Lu, Ch, Km, Qu, Is, Cy3G, Pn3G, and Pg3G were independent variables, whereas the values of L*, a*, b*, C*, and *h* were dependent variables.

### Library construction, sequencing, and data overview

Ten petal samples at five flowering stages of yellow- and purple–red-flowered tree peony cultivars were collected for isoform and RNA-seq. First, the total RNA of ten samples was extracted using the RNAprep Pure kit for plants (Tiangen, Beijing, China). RNA quality and integrity were detected using an RNA 6000 Nano Assay Kit in an Agilent Bioanalyzer 2100 system (Agilent Technologies, CA, USA). Subsequently, equal amounts of RNA from each sample were pooled together. A SMARTer PCR cDNA Synthesis Kit (Takara, Dalian, China) was applied to reverse-transcribe total RNA into cDNA. The sequencing process was conducted on a Pacific Bioscience RS II platform. Second, two petal samples at S3 from two tree peony cultivars with three biological replicates were sequenced on an Illumina HiSeq 2500 platform for comparative analysis.

Raw reads were filtered for ROIs through the Iso-seq pipeline and those with completed 5′- and 3′-cDNA primers, as well as poly A tails, were identified as FLNC transcripts. Furthermore, Iterative Clustering for Error Correction was conducted to screen consensus isoforms. High-quality full-length transcripts were confirmed under the criteria of postcorrection accuracy above 99%. ORFs of the transcripts were predicted using TransDecoder (https://github.com/TransDecoder/TransDecoder/releases). TFs were predicted by iTAK software (https://omictools.com/itak-tool)^55^. Genes were annotated based on the following databases: Nr (NCBI nonredundant protein sequences), Pfam (protein family)^56^, KOG/COG/eggNOG (Clusters of Orthologous Groups of proteins)^57,58^, Swiss-Prot (a manually annotated and reviewed protein sequence database)^59^, KEGG (Kyoto Encyclopedia of Genes and Genomes)^60^, and GO^61^.

### Differential expression analysis

Full-length transcripts sequenced by the Pacific Biosciences Sequel platform were regarded as a reference genome. RNA-seq reads of two samples were matched to the reference genome by Bowtie (v2.2.3)^62^. Quantification of gene expression was estimated by fragment per kilobase of transcripts per million mapped reads and the read counts were adjusted by the edgeR package before differential expression analysis^63^.

DEGs between two samples based on the RNA-seq results were identified using the EBSeq R package^64^. The false discovery rate (FDR) was corrected using the posterior probability values. FDR < 0.01 and |log2(fold change)| ≥ 2 were regarded as the thresholds for significant differential expression. GO enrichment analysis of DEGs was performed by the GOseq R package^65^. The statistical enrichment of DEGs in KEGG pathways was tested by KOBAS software^66^.

### qRT-PCR analysis

DEGs putatively involved in the flavonoid biosynthesis pathway were selected for qRT-PCR analysis. Total RNA was extracted with the RNAprep Pure kit for plants (Tiangen, Beijing, China) and first-strand cDNA was synthesized using the PrimeScript™ RT Master Mix reverse transcription kit (Takara, Dalian, China). After diluting the cDNA template five times to 200 ng µL^−1^, qRT-PCR was performed using TB Green TaKaRa Premix Ex Taq™ II (TaKaRa, Dalian, China) according to the manufacturer’s instructions. The reaction took place under the following conditions: denaturation at 95 °C for 15 s and 45 cycles of amplification (95 °C for 5 s, 58 °C for 30 s, and 72 °C for 31 s). *PsUbiquitin* was used as an internal reference for the expression level normalization of DEGs. Relative expression levels were calculated by the 2^−∆∆CT^ method^67^. The specific primers are listed in Supplementary Table 7. Three independent biological replicates were used in each qRT-PCR assay.

### Identification of candidate TF genes in the flavonoid pathway

Two *MYB*, two *bHLH*, and one *SPL* genes implicated in the flavonoid biosynthesis pathway were selected. Phylogeny trees of their corresponding proteins along with 101 MYBs, 94 bHLHs, and 16 SPLs from *Arabidopsis* were constructed with MEGA 6.0 using the neighbor-joining clustering method. Sequence homology alignment was performed with DNAMAN software. Structural genes related to flavonoid synthesis were screened and clustered based on their expression profiles using TB tools.

### Interaction network analysis

The interaction network was established on the basis of Pearson’s correlation coefficients, which were calculated in the *R* environment (https://www.r-project.org/); correlations with a coefficient of *R* ≥ 0.5 or *R* ≤ −0.5 were retained. The coexpressed genes with strong interconnections were considered hub genes. The relationships between candidate genes, including TF genes and structural genes, and flavonoid components were visualized by Cytoscape (v.3.7.0).

### Subcellular localization analysis of *PsMYB111*

The full-length ORF of *PsMYB111* without the termination codon was cloned into the pCAMBIA1302-GFP vector. The primers are listed in Supplementary Table 7. Subsequently, the recombinant plasmid and control pCAMBIA1302-GFP plasmid were transferred into *Agrobacterium* strain GV3101 by the freeze–thaw method. The *Agrobacterium* containing the target plasmid was resuspended in infiltration buffer (with 10 mM MES, 10 mM MgCl_2_, and 100 mM AS) to an OD600 of 0.4 and stationarily cultured for 2 h until infiltration. The *Agrobacterium* mixture was then injected into two young leaves of each *N. benthamiana* plant from the lower epidermis via a syringe without a needle. The infiltrated plants were grown in a growth chamber under the same conditions described above for 72 h, and GFP and 4′,6-diamidino-2-phenylindole fluorescence was observed under a Nikon C2-ER confocal laser scanning microscope (Nikon, Tokyo, Japan). All transient expression assays were repeated three times.

### Generation of *PsMYB111*-overexpressing tobacco

After the pCAMBIA1302-*PsMYB111* construct was transferred into the *Agrobacterium* strain GV3101, sterilized *N. tabacum* leaf disc transformation was performed following previously described methods^68^. Hygromycin and kanamycin were used to screen the transgenic tobacco lines. Rooted transgenic plants were transferred to a soil mix and grown in a greenhouse until flowering. Flowers of T2 transgenic plants were used for the detection of color indices, quantification of flavonoid levels, and qRT-PCR verification as described above. Specific primers are shown in Supplementary Table 8.

### Dual-luciferase assays

The full-length ORF of *PsMYB111* was cloned into a pGreenII62-SK vector (effector). The promoter sequences of *PsCHS* and *PsFLS* were isolated from the genomic DNA of “High Noon” using a genome walking kit (TaKaRa, Dalian, China) and then inserted into the pGreenII0800 LUC vector (reporter). All effector and reporter vectors were transformed into *Agrobacterium* strain GV3101 and isolated with kanamycin (50 mg L^−1^). Transient expression in *N. benthamiana* was conducted following the method described in subcellular localization assays. The enzyme activities of firefly luciferase (LUC) and *Renilla* luciferase (REN) were detected at 72 h after infiltration with a dual-luciferase assay system on GloMax^®^ Discover (Promega, Madison, USA). Only the promoter-LUC reporter construct with no effector was regarded as a blank control. Three independent experiments were conducted for each combination and all experiments were technically repeated three times. The specific primers used for genome walking and dual-luciferase assays are listed in Supplementary Table 9.

## Supplementary information

Supplementary Table

Supplementary Figure

## Data Availability

The raw sequence data reported in this study have been deposited in the Genome Sequence Archive (Genomics, Proteomics & Bioinformatics 2017) in the National Genomics Data Center (Nucleic Acids Res 2020), Beijing Institute of Genomics (China National Center for Bioinformation), Chinese Academy of Sciences, under accession number CRA005005, which is publicly accessible at https://bigd.big.ac.cn/gsa.
