# Supplementary material for: Integrating full-length transcriptomics and metabolomics reveals the regulatory mechanisms underlying yellow pigmentation in tree peony (Paeonia suffruticosa Andr.) flowers
Source: Hortic Res. 2021 Nov 1;8:235. doi: 10.1038/s41438-021-00666-0 (PMC8558324; doi:10.1038/s41438-021-00666-0)
Supplement: Supplementary file 1 — Supplementary Table [file 41438_2021_666_MOESM1_ESM.pdf]

Supplementary Table 1 Sequencing data statistics

Sample Name	Sample ID	cDNA size	SMRT Cells	Reads of Insert	Read Bases of Insert	Mean Read Length of Insert	Mean Read Quality of Insert	Mean Number of Passes
Tree peony	F01	1-6k	1	473,062	891,073,753	1,883	0.97	27

Supplementary Table 2 Full length sequence data

Samples	cDNA Size	Reads of Insert	Number of five prime reads	Number of three prime reads	Number of poly-A reads	Number of filtered short reads	Number of non-full-length reads	Number of full-length reads	Number of full-length non-chimeric reads	Average full-length non-chimeric read length	Full-Length non-chimeric Percentage (FLNC%)	Artificial Concatemers （%）
F01	1-6k	473,062	409,110	410,242	403,351	16,494	90,044	366,524	359,771	1,825	76.10%	1.84%

Supplementary Table 3 Clustering results by ICE

Samples	cDNA Size	Number of consensus isoforms	Average consensus isoforms read length	Number of polished high-quality isoforms	Number of polished low-quality isoforms	Percent of polished high-quality isoforms (%)
F01	1-6k	158,245	2,007	117,680	40,102	74.37%

Supplementary Table 4 Data output statistics using the SGS sequencing platform

Samples	Read Number	Base Number	GC Content	%≥Q30
High Noon'-1	23,743,135	7,083,754,008	45.18	95.29
High Noon'-2	27,363,068	8,171,858,244	45.71	94.73

High Noon'-3	26,151,034	7,817,595,230	45.35	95.15
Roufurong'-1	23,849,725	7,102,437,096	45.54	94.91
Roufurong'-2	24,209,670	7,231,381,174	44.80	95.28
Roufurong'-3	28,420,112	8,471,070,046	45.12	95.14

Supplementary Table 5 Annotation of transcript datasets to public databases

Annotated databases	Annotated number	300<=Length<1000	Length>1000
COG	24,465	3,299	21,166
GO	30,995	5,428	25,566
KEGG	26,108	4,714	21,392
KOG	36,590	5,586	31,002
Pfam	47,713	6,969	40,743
Swiss-Prot	45,865	7,282	38,581
eggNOG	55,743	9,525	46,216
Nr	56,674	9,853	46,819
All annotated	56,974	9,972	46,999

Supplementary Table 6 Genes mapped to 125 KEGG biochemical pathways

#pathway	pathway_id	Gene_number
Glycolysis / Gluconeogenesis	ko00010	598
Citrate cycle (TCA cycle)	ko00020	335
Pentose phosphate pathway	ko00030	255
Pentose and glucuronate interconversions	ko00040	282
Fructose and mannose metabolism	ko00051	222

Galactose metabolism	ko00052	270
Ascorbate and aldarate metabolism	ko00053	185
Fatty acid biosynthesis	ko00061	207
Fatty acid elongation	ko00062	91
Fatty acid degradation	ko00071	238
Synthesis and degradation of ketone bodies	ko00072	26
Cutin, suberine and wax biosynthesis	ko00073	249
Steroid biosynthesis	ko00100	93
Ubiquinone and other terpenoid-quinone biosynthesis	ko00130	102
Oxidative phosphorylation	ko00190	583
Photosynthesis	ko00195	135
Photosynthesis - antenna proteins	ko00196	49
Purine metabolism	ko00230	479
Caffeine metabolism	ko00232	10
Pyrimidine metabolism	ko00240	340
Alanine, aspartate and glutamate metabolism	ko00250	212
Glycine, serine and threonine metabolism	ko00260	283
Cysteine and methionine metabolism	ko00270	415
Valine, leucine and isoleucine degradation	ko00280	215
Valine, leucine and isoleucine biosynthesis	ko00290	88
Lysine biosynthesis	ko00300	51
Lysine degradation	ko00310	126
Arginine and proline metabolism	ko00330	281
Histidine metabolism	ko00340	58
Tyrosine metabolism	ko00350	148
Phenylalanine metabolism	ko00360	180

Tryptophan metabolism	ko00380	99
Phenylalanine, tyrosine and tryptophan biosynthesis	ko00400	181
beta-Alanine metabolism	ko00410	181
Taurine and hypotaurine metabolism	ko00430	50
Selenocompound metabolism	ko00450	127
Cyanoamino acid metabolism	ko00460	221
Glutathione metabolism	ko00480	313
Starch and sucrose metabolism	ko00500	776
N-Glycan biosynthesis	ko00510	130
Other glycan degradation	ko00511	136
Other types of O-glycan biosynthesis	ko00514	23
Amino sugar and nucleotide sugar metabolism	ko00520	458
Glycosaminoglycan degradation	ko00531	36
Glycerolipid metabolism	ko00561	204
Inositol phosphate metabolism	ko00562	191
Glycosylphosphatidylinositol(GPI)-anchor biosynthesis	ko00563	75
Glycerophospholipid metabolism	ko00564	249
Ether lipid metabolism	ko00565	83
Arachidonic acid metabolism	ko00590	51
Linoleic acid metabolism	ko00591	54
alpha-Linolenic acid metabolism	ko00592	188
Sphingolipid metabolism	ko00600	112
Glycosphingolipid biosynthesis - globo series	ko00603	22
Glycosphingolipid biosynthesis - ganglio series	ko00604	23
Pyruvate metabolism	ko00620	436
Glyoxylate and dicarboxylate metabolism	ko00630	276

Propanoate metabolism	ko00640	127
Butanoate metabolism	ko00650	84
C5-Branched dibasic acid metabolism	ko00660	40
One carbon pool by folate	ko00670	101
Carbon fixation in photosynthetic organisms	ko00710	376
Thiamine metabolism	ko00730	91
Riboflavin metabolism	ko00740	24
Vitamin B6 metabolism	ko00750	63
Nicotinate and nicotinamide metabolism	ko00760	47
Pantothenate and CoA biosynthesis	ko00770	119
Biotin metabolism	ko00780	61
Lipoic acid metabolism	ko00785	9
Folate biosynthesis	ko00790	47
Porphyrin and chlorophyll metabolism	ko00860	134
Terpenoid backbone biosynthesis	ko00900	260
Monoterpenoid biosynthesis	ko00902	59
Limonene and pinene degradation	ko00903	31
Diterpenoid biosynthesis	ko00904	9
Brassinosteroid biosynthesis	ko00905	42
Carotenoid biosynthesis	ko00906	101
Zeatin biosynthesis	ko00908	26
Sesquiterpenoid and triterpenoid biosynthesis	ko00909	67
Nitrogen metabolism	ko00910	90
Sulfur metabolism	ko00920	102
Phenylpropanoid biosynthesis	ko00940	349
Flavonoid biosynthesis	ko00941	115

Anthocyanin biosynthesis	ko00942	12
Flavone and flavonol biosynthesis	ko00944	30
Stilbenoid, diarylheptanoid and gingerol biosynthesis	ko00945	33
Isoquinoline alkaloid biosynthesis	ko00950	78
Tropane, piperidine and pyridine alkaloid biosynthesis	ko00960	87
Aminoacyl-tRNA biosynthesis	ko00970	268
Biosynthesis of unsaturated fatty acids	ko01040	140
Carbon metabolism	ko01200	1225
2-Oxocarboxylic acid metabolism	ko01210	254
Fatty acid metabolism	ko01212	367
Degradation of aromatic compounds	ko01220	23
Biosynthesis of amino acids	ko01230	1114
Vancomycin resistance	ko01502	3
ABC transporters	ko02010	68
Ribosome biogenesis in eukaryotes	ko03008	303
Ribosome	ko03010	1062
RNA transport	ko03013	746
mRNA surveillance pathway	ko03015	544
RNA degradation	ko03018	418
RNA polymerase	ko03020	132
Basal transcription factors	ko03022	142
DNA replication	ko03030	116
Spliceosome	ko03040	826
Proteasome	ko03050	255
Protein export	ko03060	144
Base excision repair	ko03410	90

Nucleotide excision repair	ko03420	161
Mismatch repair	ko03430	88
Homologous recombination	ko03440	97
Non-homologous end-joining	ko03450	24
Phosphatidylinositol signaling system	ko04070	185
Plant hormone signal transduction	ko04075	631
Ubiquitin mediated proteolysis	ko04120	474
Sulfur relay system	ko04122	31
SNARE interactions in vesicular transport	ko04130	92
Regulation of autophagy	ko04140	115
Protein processing in endoplasmic reticulum	ko04141	961
Endocytosis	ko04144	498
Phagosome	ko04145	292
Peroxisome	ko04146	324
Plant-pathogen interaction	ko04626	484
Circadian rhythm - plant	ko04712	253

Supplementary Table 7 Primers used for in-fusion cloning and qRT-PCR in tree peony.

Primers	Primer Sequences (5'-3')	Description
PsMYB4-F	ACACGGGGGACTCTTGACATGGGAAGGTCTCCTTGTTGT	In-fusion cloning
PsMYB4-R	AAGTTCTTCTCCTTTACTCTTCATCTCCAATCTTCTGTA	In-fusion cloning
PsMYB111-F	ACACGGGGGACTCTTGACATGGGGAGGGCACCCTGTTGT	In-fusion cloning
PsMYB111-R	AAGTTCTTCTCCTTTACTAGAAAGAAGCCAAGCAACCAT	In-fusion cloning
PsTT8-F	ACACGGGGGACTCTTGACATGGCTGCGCCGCCCGTAAAC	In-fusion cloning
PsTT8-R	AAGTTCTTCTCCTTTACTAGGAATTATTTGGTGTATTGC	In-fusion cloning
PsEGL3-F	ACACGGGGGACTCTTGACATGGCAACTGGGCTCCAAATT	In-fusion cloning

PsEGL3-R	AAGTTCTTCTCCTTTACTACACTTGTGAATAACTCTCTG	In-fusion cloning
PsSPL9-F	ACACGGGGGACTCTTGACATGGAAATGGGTTCGAGCGTT	In-fusion cloning
PsSPL9-R	AAGTTCTTCTCCTTTACTAAGTGACCAGTTCATCTGCTG	In-fusion cloning
PsUbiquitin-F	GACCTATACCAAGCCGAAG	qRT-PCR for tree peony
PsUbiquitin-R	CGTTCCAGCACCACAATC	qRT-PCR for tree peony
F01_cb7425_c19-F	GTAATCTGGTGGTGGTTTCGT	qRT-PCR for tree peony
F01_cb7425_c19-R	TCGTAAAGAAAGGAACCGTCA	qRT-PCR for tree peony
F01_cb9709_c126-F	ACTACCAACTCACCAAACTCC	qRT-PCR for tree peony
F01_cb9709_c126-R	CGACCAAAGAATCCAAATGAG	qRT-PCR for tree peony
F01_cb13354_c0-F	CGCAGGAGTAAGAGGTTTGGA	qRT-PCR for tree peony
F01_cb13354_c0-R	CTCTGAATATTGTTGGCCCGT	qRT-PCR for tree peony
F01_cb4550_c42-F	GAGCATGGAGGTAGAAAGAGT	qRT-PCR for tree peony
F01_cb4550_c42-R	TCCCCATTCTTTGCTTGCACT	qRT-PCR for tree peony
F01_cb4550_c24-F	CACTCTCAACCAAAACAATCC	qRT-PCR for tree peony
F01_cb4550_c24-R	CACTCTCAACCAAAACAATCC	qRT-PCR for tree peony
F01_cb4853_c44-F	AAAACCCTTCAATCCAGCTTC	qRT-PCR for tree peony
F01_cb4853_c44-R	AAATCCCCCAGTCTTCACATG	qRT-PCR for tree peony
F01_cb6443_c9-F	TTTTGTGATACTGCCCCCTCT	qRT-PCR for tree peony
F01_cb6443_c9-R	TATTTAGTGCAAAGATGGCCC	qRT-PCR for tree peony
F01_cb12671_c6-F	GTTAATGGACTCTGGGTTTGA	qRT-PCR for tree peony
F01_cb12671_c6-R	ATCCCTTTCCTCGGCATGTTT	qRT-PCR for tree peony
F01_cb7851_c6-F	CGTCTTTGGTTCTGCTGGGTT	qRT-PCR for tree peony
F01_cb7851_c6-R	ACTGCTACTTCCACCACCATT	qRT-PCR for tree peony
F01_cb8732_c11-F	AGAACAAAACCTACAACCGCC	qRT-PCR for tree peony
F01_cb8732_c11-R	ATTCCTGCTCTCTCTCTCCTC	qRT-PCR for tree peony
F01_cb8475_c68614-F	GTTCAGACAGTAGTGCCAAAG	qRT-PCR for tree peony

F01_cb8475_c68614-R	CTTCCTTATTCTCCGATCTCC	qRT-PCR for tree peony
F01_cb3984_c5-F	AAGTTCGGGTCATGGTTATTC	qRT-PCR for tree peony
F01_cb3984_c5-R	ACGTTTGTGTGTGCATCTGTT	qRT-PCR for tree peony
F01_cb8475_c28561-F	AAAAGAATTGACGAGGACCAG	qRT-PCR for tree peony
F01_cb8475_c28561-R	AGCTCCACCATTTATGCCATC	qRT-PCR for tree peony
F01_cb3692_c19-F	TCCACTCACCATCACTTCCAC	qRT-PCR for tree peony
F01_cb3692_c19-R	CTCAGACATCTCCAGTTGCAT	qRT-PCR for tree peony
F01_cb13401_c3-F	TCTTGTCACATATGCACTGCC	qRT-PCR for tree peony
F01_cb13401_c3-R	ACCCGCAGTATTTGAAGCTTA	qRT-PCR for tree peony
F01_cb9156_c0-F	CTTCATCTGCTCACTTTCAAC	qRT-PCR for tree peony
F01_cb9156_c0-R	CGGGAAAATGAAGTTGTGTTG	qRT-PCR for tree peony

Supplementary Table 8 Primers used for qRT-PCR in transgenic tobacco.

Primers	Primer Sequences (5'-3')	Description
NtTubA1-F	CTCCTATGCTCCTGTCATTTC	qRT-PCR for transgenic tobacco
NtTubA1-R	GGCGAGGATCACACTTAAC	qRT-PCR for transgenic tobacco
NtbHLH-F	GCAGCAGTGTTAGCTTGGATATG	qRT-PCR for transgenic tobacco
NtbHLH-R	AGCAGAACTCATTGAGGAAGACC	qRT-PCR for transgenic tobacco
NtSPL9-F	CTCCTTTTTGGCTTCTTGCTT	qRT-PCR for transgenic tobacco
NtSPL9-R	CTCGCTCTTGGTGGTTTCTAA	qRT-PCR for transgenic tobacco
NtC4H-F	ACAACAGAGAAAGGCGGGCAA	qRT-PCR for transgenic tobacco
NtC4H-R	AGGGGCTTTACATGACACCAA	qRT-PCR for transgenic tobacco
NtCHS-F	TGACACCCACTTGGATAGTTTAG	qRT-PCR for transgenic tobacco
NtCHS-R	CGACCTCTGGAATTGGATCAG	qRT-PCR for transgenic tobacco
NtCHI-F	CTTTTCTCGCCGCTAAATG	qRT-PCR for transgenic tobacco
NtCHI-R	TTTCTGCCACCTTCTCTG	qRT-PCR for transgenic tobacco

NtF3H-F	CAAGGCATGTGTGGATATGG	qRT-PCR for transgenic tobacco
NtF3H-R	TGTGTCGTTTCAGTCCAAGG	qRT-PCR for transgenic tobacco
NtF3'H-F	AGGCTCAACACTTCTCGT	qRT-PCR for transgenic tobacco
NtF3'H-R	CATCAACTTTGGGCTTCT	qRT-PCR for transgenic tobacco
NtFLS-F	GAACTTGAAGGGAAAAGGGGTTG	qRT-PCR for transgenic tobacco
NtFLS-R	GTAGGAGGGAGGATTTTTAGGCC	qRT-PCR for transgenic tobacco
NtDFR-F	AACCAACAGTCAGGGGAATG	qRT-PCR for transgenic tobacco
NtDFR-R	TTGGACATCGACAGTTCCAG	qRT-PCR for transgenic tobacco

Supplementary Table 9 Primers used for genome walking and Dual-luciferase assay.

Primers	Primer Sequences (5'-3')	Description
PsCHS-SP1	GGTGGGTGATTTTAGATTTGGG	Genome walking
PsCHS-SP2	CACCATGTCTTGACGTGCATCC	Genome walking
PsCHS-SP3	CTTCGGTCAGGTACATATAGCGTT	Genome walking
PsFLS-SP1	CTCTCTTCCAACTCTCTGCAAC	Genome walking
PsFLS-SP2	CCCATTCTTTGCTTGCACTGTC	Genome walking
PsFLS-SP3	TCTTTGCTTGCACTGTCGATGG	Genome walking
PsCHS-F	GTACCGGGCCCCCCCTCGAGGTCGACAGAGGAGAAGCATAGGATCGT	Dual-luciferase assay
PsCHS-R	GGCTGCAGGAATTCGATATCAAGCTTTTGTGTGTCTGGGCTCGGTT	Dual-luciferase assay
PsFLS-F	GTACCGGGCCCCCCCTCGAGGTCGACAGTGGAGTAGCAAAGGTTTG	Dual-luciferase assay
PsFLS-R	GGCTGCAGGAATTCGATATCAAGCTTGCTCTGTTTTTGAAAGCAGA	Dual-luciferase assay
PsMYB111-F	GGCCGCTCTAGAACTAGTGGATCCATGGGGAGGGCACCCTGTTG	Dual-luciferase assay
PsMYB111-R	ATCGATAAGCTTGATATCGAATTCTCAAGAAAGAAGCCAAGCAACCA	Dual-luciferase assay
