# Supplementary material for: Integrating full-length transcriptomics and metabolomics reveals the regulatory mechanisms underlying yellow pigmentation in tree peony (Paeonia suffruticosa Andr.) flowers
Source: Hortic Res. 2021 Nov 1;8:235. doi: 10.1038/s41438-021-00666-0 (PMC8558324; doi:10.1038/s41438-021-00666-0)
Supplement: Supplementary file 2 — Supplementary Figure [file 41438_2021_666_MOESM2_ESM.doc]

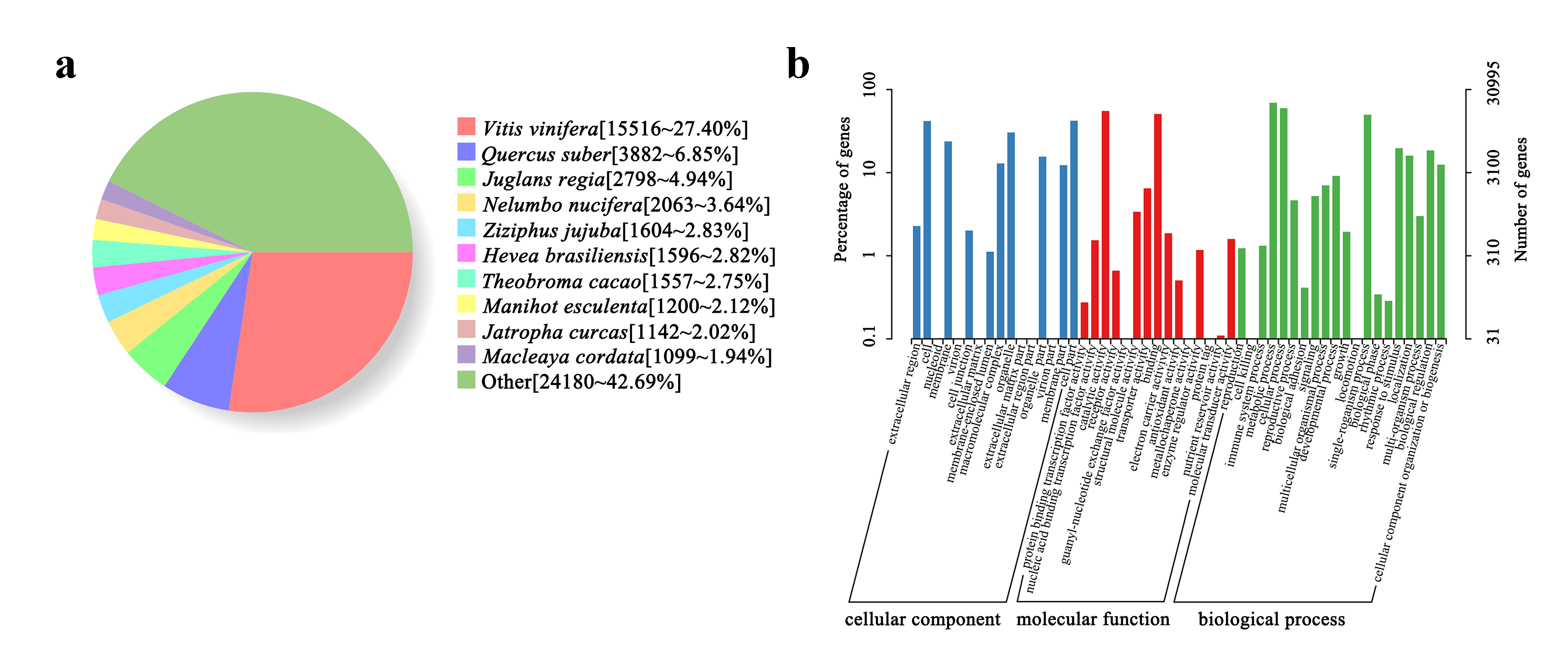


**Supplementary Fig. 1 Functional annotation of transcripts.** **a** Nr Homologous species distribution diagram of transcripts. **b** Distribution of GO terms for all annotated transcripts.


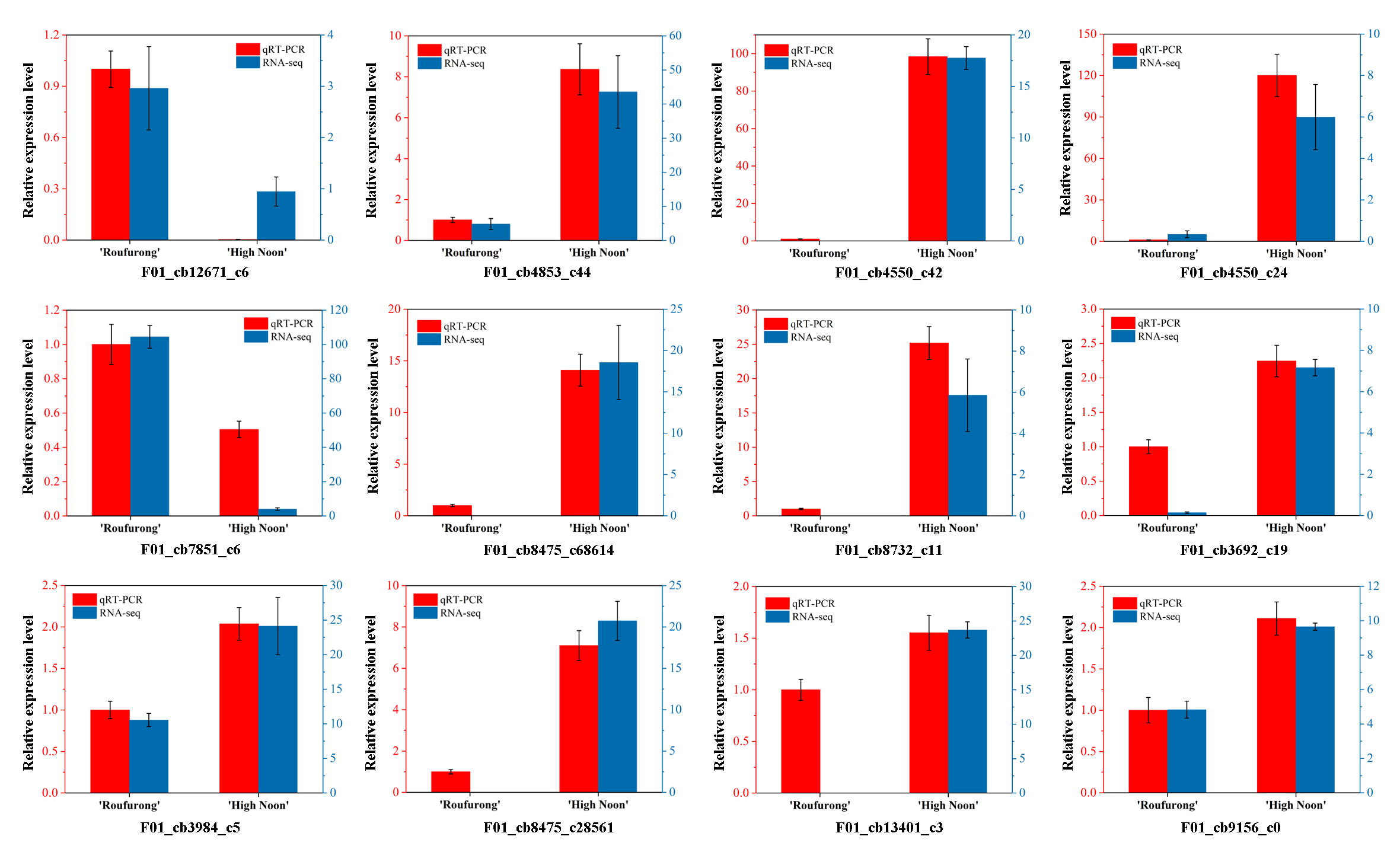


**Supplementary Fig. 2 qRT-PCR verified the flavonoid biosynthesis related differentially expressed genes (DEGs).** The floral samples from tree peony cultivars ‘High Noon’ and ‘Roufurong’ at S3 were used for expression analysis. Standard error (SE) of the mean from three biological replicates is shown as error bars.


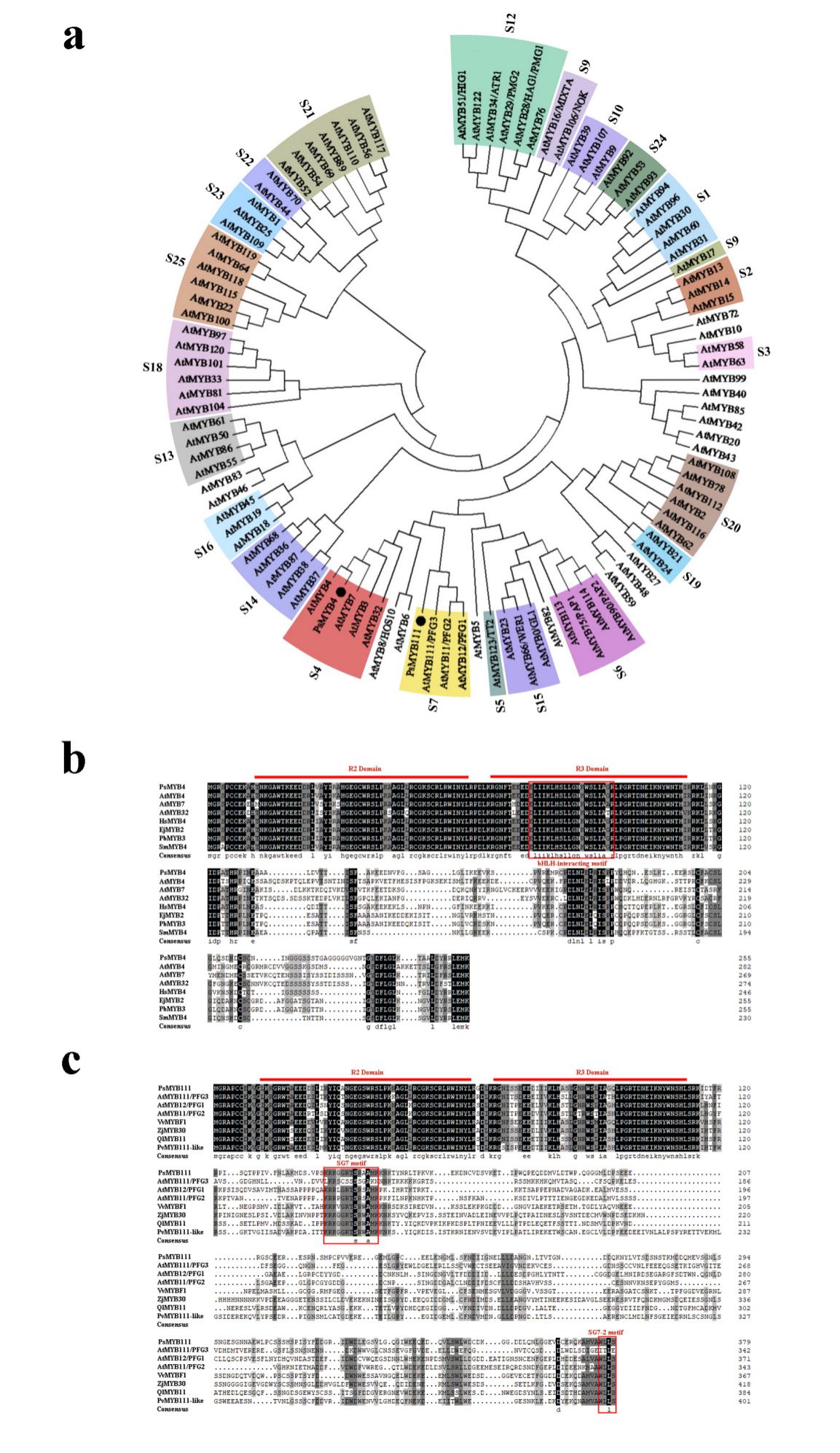


**Supplementary Fig. 3 Phylogenetic analysis of PsMYB4 and PsMYB111 with their homologous proteins. a** Phylogenetic analyses of PsMYB4 and PsMYB111 in Arabidopsis. **b** Multiple alignments of deduced amino acid sequences of the PsMYB4 protein with other species. **c** Multiple alignments of deduced amino acid sequences of the PsMYB111 protein with other species.


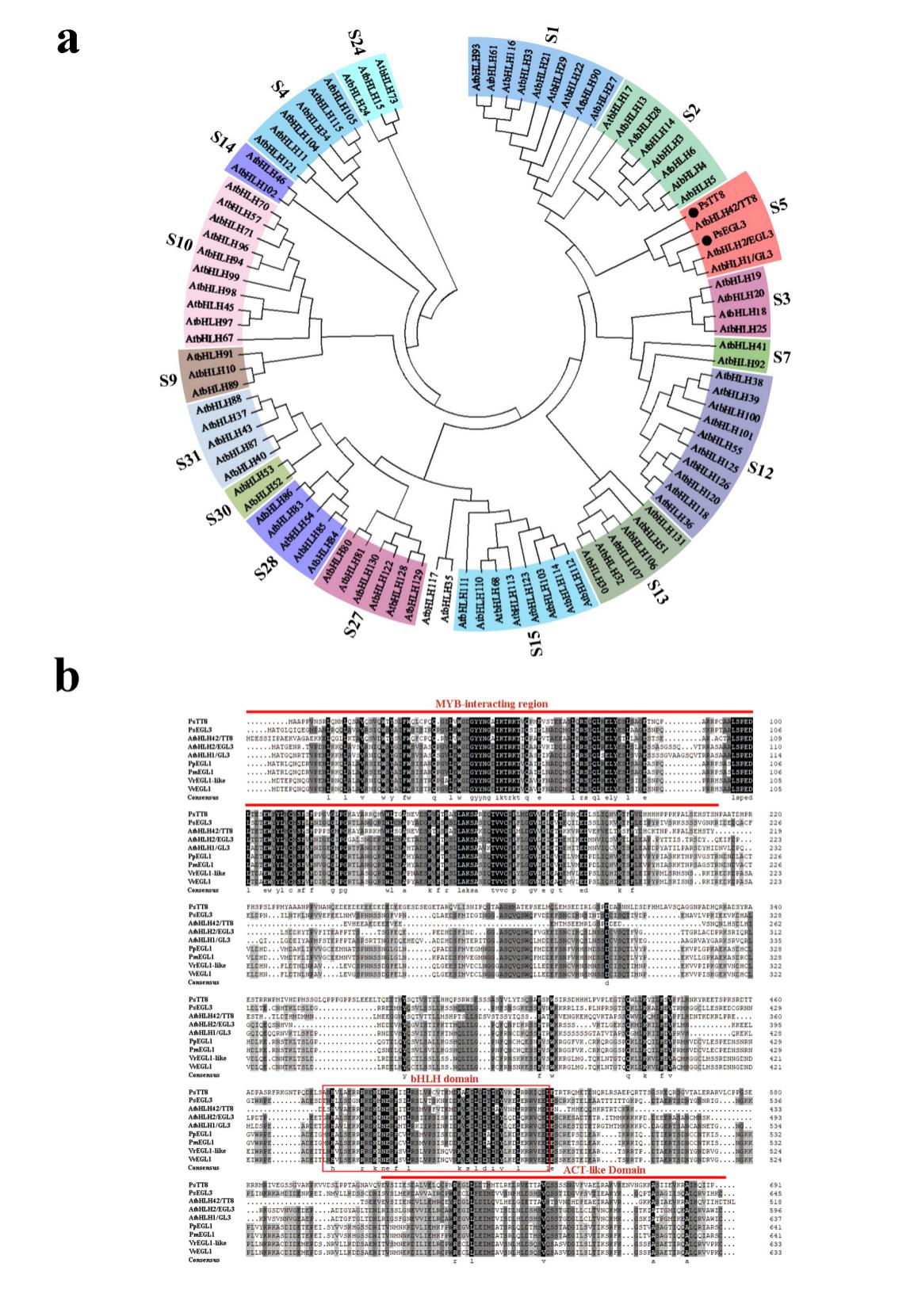


**Supplementary Fig. 4 Phylogenetic analysis of PsTT8 and PsEGL3 with their homologous proteins. a** Phylogenetic analyses of PsTT8 and PsEGL3 in Arabidopsis. **b** Multiple alignments of deduced amino acid sequences of the PsTT8 and PsEGL3 proteins with other species.


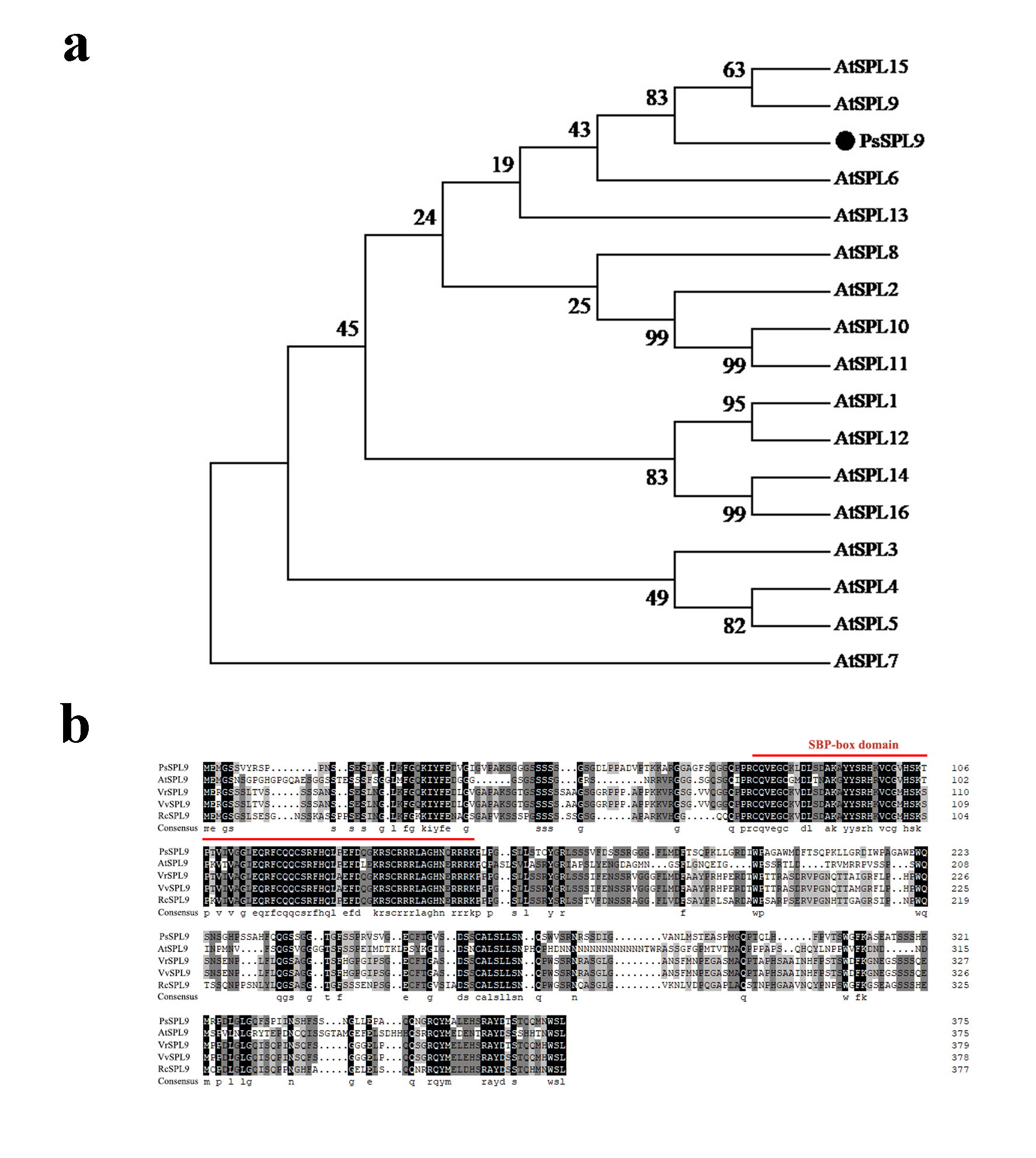


**Supplementary Fig. 5 Phylogenetic analysis of PsSPL9 and its homologous proteins. a** Phylogenetic analysis of PsSPL9 in Arabidopsis. **b** Multiple alignments of deduced amino acid sequences of the PsSPL9 protein with other species.
